# Changes in the Temporal Targeting of the U.S. National Ambient Air Quality Standards (NAAQS) for SO_2_ Reduce Average and Peak Emissions from Coal Power Plants

**DOI:** 10.1021/acs.est.4c10718

**Published:** 2025-06-18

**Authors:** Joanna H. Slusarewicz, Valerie J. Karplus

**Affiliations:** † Department of Engineering and Public Policy, Carnegie Mellon University, 5000 Forbes Ave, Pittsburgh, Pennsylvania 15213, United States; ‡ Wilton E. Scott Institute for Energy Innovation, Carnegie Mellon University, Pittsburgh, Pennsylvania 15213, United States

**Keywords:** environmental regulation, SO_2_ emissions, pollution monitoring, coal power plants

## Abstract

The U.S. National Ambient Air Quality Standards (NAAQS) protect public health by limiting ambient pollutant concentrations, but effects at power plants are not well characterized. We estimate how a 2010 SO_2_ NAAQS change that altered policy targeting from peak hourly emissions to annually aggregated hourly emissions affected SO_2_ emissions from coal-fired power plants. Using data on electricity generating unit (EGU) characteristics, SO_2_ emissions, fuel prices, and PM_2.5_ NAAQS classifications from 2001 to 2019 from public data sources, we estimate that, after a county was classified under the 2010 SO_2_ standard, EGUs reduced SO_2_ emissions during daily maximum hours by 31.6% (95% CI [−0.381, −0.247]) at the 99th percentile and 34.4% (95% CI [−0.424, −0.253]) at the 50th percentile. After a nearby ambient SO_2_ monitor was added to assess NAAQS compliance, hourly emissions fell by 12.4% (95% CI [−0.188, −0.054]) at the 99th percentile and 14.4% (95% CI [−0.228, −0.050]) at the 50th percentile. Our results also suggest the new standard had the same effect on peak hourly SO_2_ emissions as median and that plants in counties prioritized for early designation were less responsive to the policy change. These results suggest that the 2010 SO_2_ NAAQS change may have reduced SO_2_ emissions but may not have had an outsized impact on peak emissions despite policy guidance encouraging their control.

## Graphical abstract

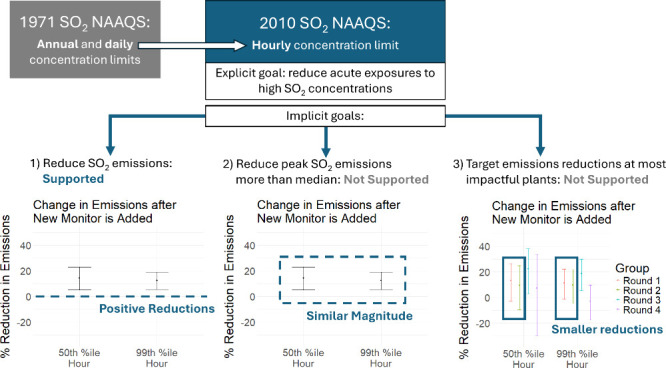

## 1 Introduction

Sulfur dioxide (SO_2_) emissions from coal-fired power plants are a major source of public health damage in the United States. High SO_2_ concentrations exacerbate respiratory disease and cause severe impairment or mortality in at-risk populations.^1,2^ Atmospheric SO_2_ also contributes to the formation of particulate matter, which impairs respiratory health.^3^ Most atmospheric SO_2_ originates from the industrial and electricity sectors, where coal-fired power plants make up 60% of U.S. SO_2_ emissions.^2^

To protect public health, the EPA limits outdoor concentrations of air pollutants through the U.S. Clean Air Act’s National Ambient Air Quality Standards (NAAQS). These standards are set nationally, after which states develop monitoring and modeling strategies based on the location of major pollution sources and population centers and implement these strategies to assess regional NAAQS compliance.^4^ States respond to subsequent attainment designations by issuing State Implementation Plans (SIPs) that describe how they will maintain or achieve attainment. For example, for SO_2_, nonattainment SIPs typically require major sources to install controls or change fuel type.^5^ The state-level standards are intended to deter polluters and ultimately improve or protect regional air quality.

Medical evidence indicating public health risks from acute periods of high SO_2_ concentrations^6^ led the EPA to change the annual and daily SO_2_ NAAQS to an hourly standard in 2010.^7^ The 2010 SO_2_ NAAQS change went beyond previous NAAQS revisions for other pollutants by abandoning the existing annual average and maximum-daily average standards in favor of a limit on the 99th percentile of 1-h daily maximum concentrations.^8^ This change both functionally increased the stringency of the standard and changed the target of the standard to encourage reductions in peak SO_2_ concentrations.

The change in temporal unit also required states to expand their monitoring and modeling capabilities to accurately detect periods of high SO_2_ concentrations.^4^ This expansion followed a decades-long decline in national ambient monitoring capacity from around 1,500 ambient SO_2_ monitors in 1980 to around 450 in 2013, with the most recent declines attributed to “increasingly limited resources at the local, state, and federal levels”.^4^ Many areas either had insufficient ambient monitoring coverage to determine compliance or existing monitors only suitable for longer aggregation periods.

To give states time to develop models and install monitoring equipment, the EPA staggered its implementation of the NAAQS. The EPA assigned each county to one of four NAAQS implementation rounds based on the need for new monitoring and risk of nonattainment.^9,10^ A county’s round determined the first year it was subject to the 2010 SO_2_ NAAQS standards rather than the 1972 standard: round 1 counties were first designated in 2013, round 2 in 2016, round 3 in 2018, and round 4 in 2020. A county would only be designated in round 1 if it already had sufficient monitoring capacity to evaluate compliance with the new hourly standard, meaning that round 1 counties may have already been under greater policy pressure before the NAAQS change. Counties were designated in round 2 if they contained coal electricity generating units (EGUs) with large quantities of aggregate emissions or moderate annual emissions and relatively high emissions rates. Otherwise, counties were assigned to rounds 3 or 4. Table 1 summarizes the inclusion criteria for counties in each round, the year in which they were first assessed for attainment under the 2010 standard, and the number of EGUs in counties designated during that round.

**Table 1:** Initial Designation Year, Round Inclusion Criteria, and Number of Contained Coal EGUs for Counties in Each Designation Round of the 2010 SO_2_ NAAQS

			# of Affected Coal Units	
Round	Year	Description	Unbalanced Data	Balanced Data
1	2013	Areas with hourly SO_2_ monitoring data from 2009 to 2011 ^11^	139	45
2	2016	Areas with newly detected violations or with SO_2_ sources with (a) annual SO_2_ emissions more than 16,000 tons or (b) annual SO_2_ emissions more than 2,600 tons and an annual SO_2_ emissions rate of more than 0.45 lbs/mmBtu^12–14^	188	130
3	2018	Most remaining areas, primarily using modeled concentration data^15,16^	811	263
4	2020	Areas choosing to implement new monitoring rather than modeling^17,18^	38[Table-fn t1fn2]	18[Table-fn t1fn2]

a The low number of round 4 counties may result from the fact that counties at a low risk of violation preferred modeling as a less expensive alternative to monitoring.

The expansion in monitoring capacity required by the 2010 SO_2_ NAAQS change placed additional restrictions on already strained state budgets, but the benefits gained from these additional monitors are not yet well understood. The EPA estimated that if all US counties were brought into attainment under the 2010 SO_2_ standard, it would create $14 to $33 billion in net benefits (in 2006 dollars) by 2020 by reducing acute SO_2_ exposures and PM formation. In the same report, the new standard’s estimated cost to industry was projected to be around $1 billion, and the cost of maintaining monitoring networks was estimated to be $15 million annually.^2^ Quantifying the effect of the NAAQS on SO_2_ emissions is an important step toward understanding industry responses and evaluating costs *ex post*.

Evidence from existing studies on the effects of air quality standards on pollution concentrations is mixed. Some studies suggest minimal effects of NAAQS on pollution concentrations, based on analysis of the effect of attainment status^19^ or EPA enforcement actions^20^ on air quality outcomes. Others show that various state standards were at least partially effective in reducing industrial emissions.^20–24^ However, to the best of our knowledge, no study investigates the relationship between changes in NAAQS and changes in emissions at large point-source emitters, such as coal power plants. This analysis addresses this gap by assessing the effect of the NAAQS on both the quantity and temporal distribution of emissions and using hourly emissions data to estimate the effect of the 2010 SO_2_ NAAQS update on polluters directly.

This study also expands upon prior studies of NAAQS implementation by distinguishing the *stringency* of the SO_2_ standard from the *unit of measurement*. Our study first estimates whether the increase in standard stringency was effective at reducing annual average SO_2_ emissions. Then, we examine whether the 2010 SO_2_ NAAQS change ultimately resulted in peak hourly emissions reductions beyond what may have been achieved by reducing the existing daily and annual concentration limits. Our estimation method is limited by the heterogeneous implementation of the 2010 NAAQS across plants based on pre-existing emissions, but we take several steps to address possible endogeneity. The effect on SO_2_ emissions from coal EGUs is identified using an empirical strategy that disentangles the effect of the 2010 SO_2_ NAAQS from simultaneous policy and economic shocks by using the placement of monitors rather than policy promulgation as a measure of enforcement. We also estimate the effects of 2010 SO_2_ NAAQS on different groups of plants based on the date that their counties were first designated. We use this by-round heterogeneity to estimate whether the NAAQS change had a greater effect on the plants that were included in earlier implementation rounds.

## 2 Data and Methods

### 2.1 Data Sources

Data for this analysis were compiled from several sources. Regression analysis is run using a balanced panel of coal-fired EGUs, excluding units that began operating or closed down over the period of 2001–2019. The results therefore estimate the effect of the NAAQS on emissions from plants that remain open, i.e., focusing on the intensive margin, and do not capture emissions reductions from plant retirements or refueling to natural gas induced by the policy change. In addition, the exclusion of retiring EGUs means that the estimated effect of the NAAQS is only the effect on plants that remained operational and does not include any effect that the NAAQS change may have had on the emissions of retired plants via operational changes in the years before they exited the sample. EGUs that retired before 2019 may have had different characteristics compared to plants that remained in operation, and therefore, their preretirement emissions may have responded differently to the NAAQS change. Thus, the results should be interpreted as a partial effect of the policy change conditional on survival over the entire period and, thus, likely a conservative estimate of emissions changes.

#### SO_2_ Emissions Metrics

EGUs may respond in different ways to SO_2_ emission limits in ways that affect different measures. Therefore, four dependent variables are used to capture changes in SO_2_ emissions, each calculated per EGU per year: (1) total emissions (in tons), (2) annual emissions rate (in g/kWh), (3) the 99th percentile of daily maximum emissions hours (in lbs), and (4) the median of daily maximum emissions hours (in lbs). These measures along with annual production were obtained from the Clean Air Markets Program Data set^25^ published by the EPA.

#### 2010 SO_2_ NAAQS Policy Indicator

Since the SO_2_ NAAQS change was implemented in four separate rounds, one policy indicator is set equal to 1 in and after the first year an EGU’s county was designated under the standard. In addition, since EGUs may have responded differently based on their county’s designation round, an additional regression is run with the policy indicator interacted with implementation round dummy variables. Both measures were obtained by coding each county based on the annual designations published in the Federal Register.^11,13–18^

The number of 5-min duration SO_2_ monitors added within a 50 km radius of the EGU since 2010 is used as a policy measure that overcomes some of the weaknesses of the implementation indicator. Monitor installations reflect state efforts to assess attainment under the new standard, which required greater precision and data completeness than the more aggregated standards. Therefore, the variable “# monitors” captures a specific dimension of NAAQS policy implementation. Since monitor additions were more heterogeneous in time across EGUs and rounds, this policy variable partially overcomes the correlation between policy treatment date and EGU emissions. Furthermore, while the designation date indicator identifies the effect of national policy enforcement, ambient monitor installation is a result of state-level implementation efforts. The effect of the number of new ambient SO_2_ monitors is therefore less sensitive to heterogeneity in state enforcement since it represents the effect of the SO_2_ NAAQS change on plants conditional on state implementation.

Monitor data were obtained from the Air Quality System Database^26^ which contains a list of all monitors used by the EPA to assess NAAQS compliance, including their locations and operating years. The 50 km radius was chosen because the EPA permits state agencies to assess SO_2_ NAAQS compliance with monitors that cover an “urban scale”, a spatial scale covering up to 50 km.^27^ Additional regressions that consider the effect of monitors added at the neighborhood scale (within 4 km) are included in the Supporting Information.

#### Additional Variables

To distinguish the effects of the 2010 SO_2_ NAAQS policy change from other NAAQS changes, controls are included for the 2006 and 2012 PM_2.5_ NAAQS attainment status. These standards were also updated over the time period though they did not require additional monitoring to enforce. They also indirectly capture some of the variation in regulatory oversight, since nonattainment counties are more heavily targeted by regulatory efforts. The PM_2.5_ attainment status variable was equal to 1 in every year a county was designated nonattainment under the PM_2.5_ NAAQS. Status was determined for each year from 2001 to 2019 based on county designations published by the EPA.^28^ The effect of the 2010 NO_*x*_ NAAQS change was also considered, but no coal-fired EGUs were ever in nonattainment counties.

Other national policy changes over the time period include the Mercury and Air Toxics Standards (MATS) and the Cross-State Air Pollution Rule (CSPAR). Instead of acting on plants through state enforcement of air quality standards, these policies set emissions rate limits directly on plants nationwide. Since these policies regulated emissions and not air quality, these policies would not require the installation of additional ambient SO_2_ monitors. This analysis relies on the spatiotemporal variability of the 2010 SO_2_ NAAQS implementation and ambient monitor installations to distinguish its effects separately from these policies.

To account for the changing fuel prices over this period, a control is included for the ratio of the national average coal price to the state average natural gas price on an energy basis. The national average coal price is the average of all prices paid by coal plants each year and is obtained from the EIA.^29^ These data are only fully available at the national level of aggregation. However, the EIA does provide state-level average citygate natural gas prices for all states,^30^ which are used to construct a state level economic indicator.

Finally, all regressions include EGU fixed effects to control for characteristics of the EGUs that affect emissions and do not change over time. These fixed effects also absorb the effects of the time-invariant components of state-level policy enforcement, grid demand profiles, and concurrent pre-2000 policy requirements faced by EGUs.

One concern regarding identification of the policy effect is whether monitor placement is correlated with unit emissions and, more problematically, with unit responsiveness to policy intervention. One strategy used to address this concern was to absorb any time-invariant heterogeneity between units that did and did not have monitors installed nearby in EGU-level fixed effects. Furthermore, heterogeneous effects will be partially accounted for since the EPA defined 2010 SO_2_ implementation round by specifically identifying high-priority EGUs for round 1 or 2 designation. Monitor placement is therefore more likely to be exogenous to EGU characteristics within each implementation round.

### 2.2 Regression Setup

The 2010 SO_2_ NAAQS for hourly SO_2_ emissions served as a more stringent limit on daily and annual concentrations. Furthermore, the 2010 SO_2_ NAAQS guidance advises that coal plants install SO_2_ control technologies to reduce emissions.^31^ This would generally decrease emissions at all hours as a factor of the scrubber’s efficiency, thereby reducing aggregate emissions at the daily and annual level. Indeed, if the policy change was effective, we would expect an EGU’s SO_2_ emissions to decrease for both hourly and annual measures, motivating the first hypothesis: *The implementation of the 2010 SO*_*2*_ *NAAQS is correlated with a decrease in an EGU*’*s SO*_*2*_ *emissions and emissions intensity after controlling for national fuel price changes, coincident EPA regulatory actions, and EGU fixed effects.*

While the 2010 SO_2_ NAAQS functionally placed stricter limits on overall SO_2_ concentrations, the stated target of the standard was to reduce peak concentrations. If the new standard was indeed more effective at targeting peak emissions than the existing standard, then peak emissions would decrease more than proportionally with median emissions. Otherwise, the same effects could have been achieved by strengthening the existing standard without added regulatory uncertainty and monitoring burden. The second hypothesis investigates whether the 2010 standard change effectively motivated reductions in peak emissions beyond reductions made on average: *The estimated effect of the policy variable on the 99* ^*th*^ *percentile of maximum daily SO*_*2*_ *emissions hours will be negative and larger proportionally than the impact on median or maximum daily SO*_*2*_ *emissions hours.*

Eqs 1 and 2 below test hypotheses #1 and #2 using the effect of the 2010 SO_2_ NAAQS policy implementation on a balanced panel of EGUs operating using coal from 2001 to 2019.

1 $$\ln{\left(E\right)}_{i,t}=\alpha_{0}+\beta_{C}C_{c,t}+\beta_{P}P_{s,t}+\beta_{M^{06}}M_{c,t}^{06}+\beta_{M^{12}}M_{c,t}^{12}+\gamma_{i}+\epsilon_{i,t}$$

Here, *E* represents one of four measures of EGU emissions behavior for EGU *i* in year *t*: the gross annual SO_2_ emissions, the average SO_2_ emissions rate, and the 99th and 50th percentiles of daily maximum hourly SO_2_ emissions. The variable of interest is *C*_*c*,*t*_, a county-level indicator variable which equals 1 when the county *c* was designated under the 2010 SO_2_ NAAQS. Coefficient β_*C*_ thus represents the relative difference in emissions before and after the policy was implemented in the EGU’s county.

We include several regressors to control for possible confounding economic and policy influences on SO_2_ emissions. Coefficient β_*P*_ describes the relationship between SO_2_ and *P*_*s*,*t*_ which is the average citygate price of natural gas in state *s* over the average nationwide price of coal during year *t*. Coefficients β_*M* ^06^_ and β_*M* ^12^_ describe the effect of the designation of the county under the 2006 and 2012 PM_2.5_ standards, respectively. Finally, γ_*i*_ represents the EGU-level fixed effects. The regression uses robust standard errors clustered at the facility level, where the facility is a power plant containing one or more EGUs.

This analysis also considers changes in emissions associated with the installation of new ambient monitors. The following equation tests hypotheses #1 and #2 using new monitor additions after 2010 as the policy indicator:

2 $$\ln{\left(E\right)}_{i,t}=\alpha_{0}+\beta_{A}A_{i,t}+\beta_{P}P_{s,t}+\beta_{M^{06}}M_{c,t}^{06}+\beta_{M^{12}}M_{c,t}^{12}+\gamma_{i}+\epsilon_{i,t}$$

This replaces the primary measure of the policy effect in eq 1 with *A*_*i*,*t*_, the number of 5 min SO_2_ ambient monitors installed within 50 km since 2010 for EGU *i* in year *t*. Therefore β_*A*_ represents the average effect of adding each additional *SO*_2_ ambient monitor on EGU emissions.

Both eqs 1 and 2 test hypothesis #1 through the magnitude and sign of the policy coefficient for all measures of SO_2_ emissions. Negative coefficients indicate that the policy was associated with lower emissions. Meanwhile, hypothesis #2 is tested by comparing coefficients when *ln*(EGU SO_2_) is the 99th or 50th percentile of maximum daily hourly emissions. Hypothesis #2 is supported if the 99th percentile coefficient is significantly less than the 50th percentile coefficient.

It should be noted that counties’ initial designation dates varied based on the existence of hourly SO_2_ concentration data and the priority level placed on nearby emissions sources.^10^ Regions already subject to greater policy scrutiny would have monitoring data that allowed for round 1 compliance assessment, and regions containing plants qualifying as “high priority” by the EPA were designated in round 2. The implication is that EGUs located in areas classified during rounds 3 and 4 were not considered high priority by state or national regulators. The following hypothesis captures the expectation that regulators may have placed more pressure on plants in round 1 and 2 counties to reduce emissions since they were either already subject to additional policy scrutiny or were considered high-risk of causing violations: *The relationship between the 2010 SO*_*2*_ *NAAQS implementation and EGU emissions differs across rounds, controlling for EGU fixed effects, coal over gas price, and county PM*_*2.5*_ *classification.* According to this hypothesis, EGUs located in counties designated in rounds 1 and 2 would see greater reductions in SO_2_ emissions compared with those in counties designated during rounds 3 or 4.

Hypothesis #3 is tested by comparing policy effects by round. Eqs 3 and 4 follow from eqs 1 and 2 but include fixed effects for the policy round as δ_*R*_.

3 $$\ln{\left(E\right)}_{i,t}=\alpha_{0}+\delta_{R}\times \beta_{C}C_{c,t}+\beta_{P}P_{t}+\beta_{M^{06}}M_{c,t}^{06}+\beta_{M^{12}}M_{c,t}^{12}+\gamma_{i}+\epsilon_{i,t}$$

4 $$\ln{\left(E\right)}_{i,t}=\alpha_{0}+\delta_{R}\times \beta_{A}A_{i,t}+\beta_{P}P_{t}+\beta_{M^{06}}M_{c,t}^{06}+\beta_{M^{12}}M_{c,t}^{12}+\gamma_{i}+\epsilon_{i,t}$$

δ _*R*_ is not treated as a fixed effect since the variation would be absorbed by EGU-level fixed effects. Instead, δ_*R*_ is interacted with the policy variable, which is the policy implementation indicator in eq 3 or the new monitor count variable in [Disp-formula eq4]. The interaction shows whether the policy’s effect on SO_2_ emissions is different between, which may occur if EGUs in different rounds are subject to different levels of policy pressure.

## 3 Results

### 3.1 Relationship between NAAQS Implementation and SO_2_ Emissions

The first columns of each dependent variable in Table 2 show the coefficients for 2010 SO_2_ NAAQS implementation estimated from eq 1, and the first columns in Table 3 show the coefficients for the number of new nearby ambient SO_2_ monitor installations estimated from eq 2. These tables show that both measures of policy implementation were associated with statistically significant average SO_2_ emissions reductions across all metrics at the *p* = 0.05 level. Figure 1 shows the percentage reductions in SO_2_ emissions associated with the coefficients for each policy variable, where Panel A shows that the magnitude of the policy coefficient is largest for annual SO_2_ emissions, corresponding to a 50.7% drop (95% CI [−0.574, −0.430]). Panel B shows that the coefficients associated with the new post-2010 monitor count are also most negative for annual emissions quantity, corresponding to a 20.7% drop (95% CI [−0.292, −0.111]). These results indicate that the SO_2_ NAAQS implementation may have contributed to reductions in SO_2_ emissions. This policy-induced reduction could indicate that EGUs reduced either the rate of SO_2_ emissions or the quantity of electricity produced in response to the policy.

**Figure 1:**
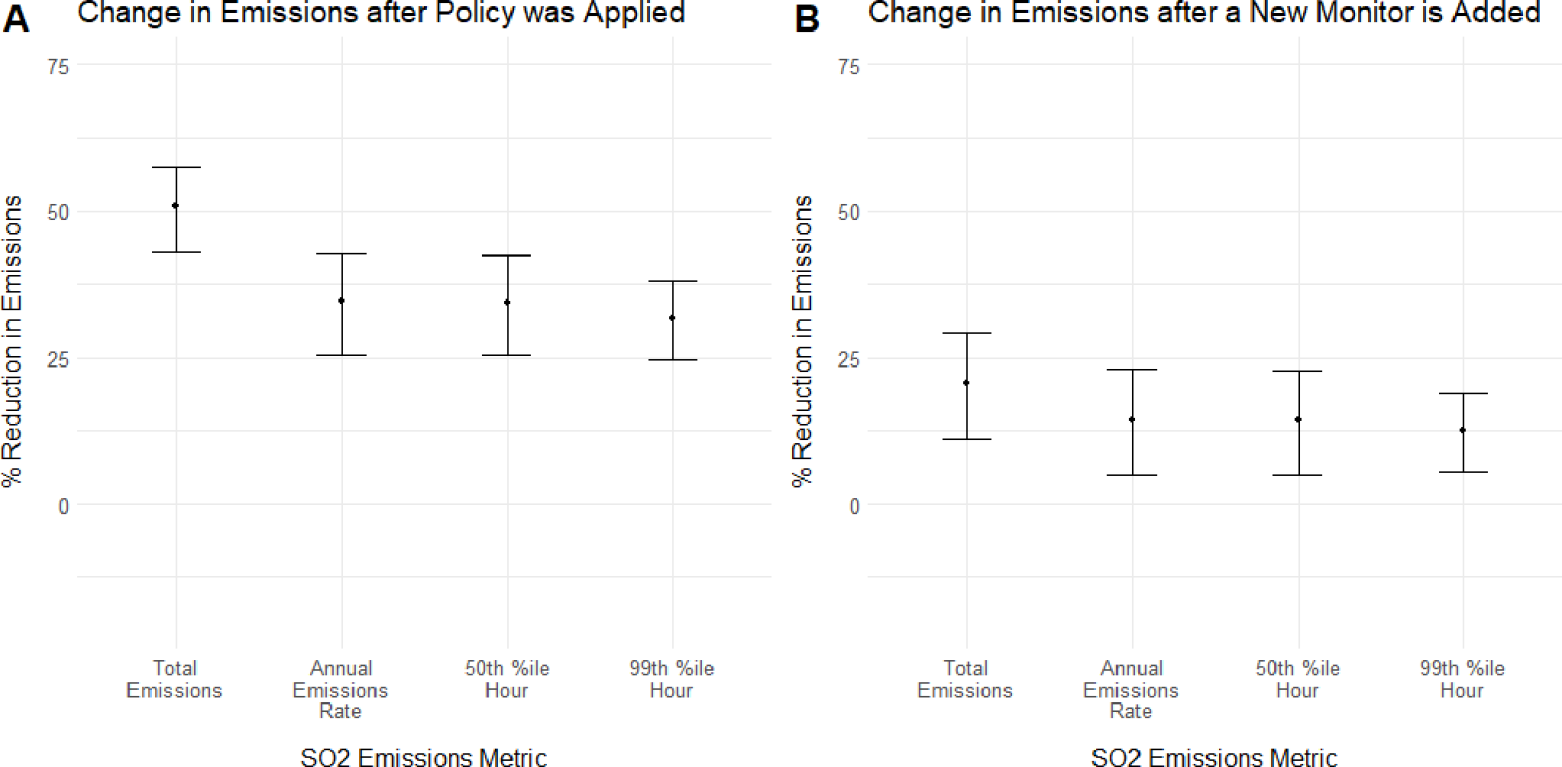
Policy-associated emissions reductions. For both the policy indicator and post-2010 monitor count variables, there were statistically significant emissions reductions for all measures of emissions. Note: Plot A shows the estimated amounts by which emissions are lower for a year after the policy was implemented in an EGU’s county as opposed to a year before, which are calculated from eq 1. Plot B shows the estimated amount that emissions will be lower for an EGU in a year after a new monitor is installed nearby, calculated from the results of regression 2. Reductions are shown for the regression run with each of the four measures of emissions. Error bars are calculated using robust standard errors clustered at the power plant level. Regressions only include EGUs in the balanced data set (i.e., in operation for all years between 2001 and 2019).

**Table 2:** Resulting Coefficient Estimates from Regressions of Each of the Four Unit Emissions Measures on a Dummy Variable Indicating Whether the 2010 SO_2_ NAAQS Had Been Implemented in the Unit’s County[Table-fn t2fn5]

	*Dependent variable:*							
	log(Annual SO_2_ (tons))		log(Annual SO_2_ Rate (g/kWh))		log(99%ile SO^2^ h (lbs))		log(50%ile SO_2_ h (lbs))	
Policy Flag	–0.716^d^		–0.427^d^		–0.384^d^		–0.424^d^	
	(0.074)		(0.067)		(0.049)		(0.066)	
Policy Flag (Round 1)		–0.389		–0.203		–0.251^c^		–0.189
		(0.256)		(0.177)		(0.119)		(0.183)
Policy Flag (Round 2)		–0.517^d^		–0.320^c^		–0.251^c^		–0.329^c^
		(0.150)		(0.153)		(0.113)		(0.143)
Policy Flag (Round 3)		–1.003^d^		–0.599^d^		–0.543^d^		–0.589^d^
		(0.092)		(0.073)		(0.065)		(0.075)
PM2.5 Nonattainment (2006)	–0.176	–0.172	–0.114	–0.110	0.096	0.095	–0.243	–0.238
	(0.244)	(0.252)	(0.175)	(0.178)	(0.128)	(0.129)	(0.189)	(0.192)
PM2.5 Nonattainment (2012)	–0.333^d^	–0.450^b^	–0.014	–0.098	–0.116	–0.159	0.121	0.033
	(0.128)	(0.249)	(0.229)	(0.312)	(0.100)	(0.152)	(0.078)	(0.128)
Gas/Coal Price	0.468^d^	0.473^d^	0.360^d^	0.363^d^	0.253^d^	0.255^d^	0.373^d^	0.376^d^
	(0.035)	(0.036)	(0.031)	(0.032)	(0.021)	(0.021)	(0.032)	(0.033)
Constant	7.234^d^	7.250^d^	–0.621^d^	–0.613^d^	7.844^d^	7.854^d^	6.603^d^	6.611^d^
	(0.109)	(0.108)	(0.099)	(0.098)	(0.064)	(0.063)	(0.100)	(0.100)
Observations	8,778	8,778	8,778	8,778	8,778	8,778	8,772	8,772
R^2^	0.640	0.644	0.589	0.591	0.648	0.650	0.621	0.622
Adjusted R^2^	0.620	0.624	0.566	0.568	0.629	0.631	0.600	0.601

a *p* < 0.1.

b *p* < 0.05.

c *p* < 0.01.

d Other independent variables represent the county’s PM_2.5_ status with respect to the 2006 and 2012 standards and the national gas/coal unit price. For each dependent variable, the first column shows the results of eq 1, and the second column shows the results of eq 3. Unit-level fixed effects are included. Note that round 4 counties were not classified under the policy until after 2019, so there are no estimates provided for the effect of policy implementation for units in these counties.

**Table 3:** Resulting Coefficient Estimates from Regressions of Each of the Four Unit Emissions Measures on New Ambient SO_2_ Monitors Added within 50 km of the Unit since 2010[Table-fn t3fn5]

	*Dependent variable:*							
	log(Annual SO_2_ (tons))		log(Annual SO_2_ Rate (g/kWh))		log(99%ile SO_2_ h (lbs))		log(50%ile SO_2_ h (lbs))	
Post-2010 New Monitor Count	–0.231^d^		–0.155^d^		–0.132^d^		–0.155^d^	
	(0.058)		(0.054)		(0.039)		(0.053)	
Post-2010 New Monitor Count (Round 1)		–0.202^b^		–0.150^b^		–0.119^b^		–0.140
		(0.121)		(0.082)		(0.067)		(0.085)
Post-2010 New Monitor Count (Round 2)		–0.108		–0.094		–0.100		–0.097
		(0.094)		(0.104)		(0.075)		(0.096)
Post-2010 New Monitor Count (Round 3)		–0.434^d^		–0.263^c^		–0.205^d^		–0.252^c^
		(0.128)		(0.116)		(0.077)		(0.115)
Post-2010 New Monitor Count (Round 4)		–0.066		0.042		0.031		–0.074
		(0.142)		(0.100)		(0.067)		(0.172)
PM2.5 Nonattainment (2006)	–0.062	–0.051	–0.054	–0.048	0.153	0.158	–0.184	–0.179
	(0.226)	(0.223)	(0.174)	(0.174)	(0.124)	(0.122)	(0.187)	(0.187)
PM2.5 Nonattainment (2012)	–0.556^c^	–0.584^c^	–0.145	–0.155	–0.234	–0.245	–0.008	–0.022
	(0.231)	(0.246)	(0.294)	(0.298)	(0.157)	(0.165)	(0.094)	(0.100)
Gas/Coal Price	0.502^d^	0.496^d^	0.377^d^	0.374^d^	0.269^d^	0.267^d^	0.389^d^	0.386^d^
	(0.035)	(0.035)	(0.032)	(0.032)	(0.021)	(0.021)	(0.032)	(0.032)
Constant	7.056^d^	7.072^d^	–0.718^d^	–0.709^d^	7.754^d^	7.759^d^	6.509^d^	6.517^d^
	(0.109)	(0.108)	(0.099)	(0.098)	(0.066)	(0.066)	(0.099)	(0.099)
Observations	8,778	8,778	8,778	8,778	8,778	8,778	8,772	8,772
R^2^	0.628	0.632	0.584	0.586	0.642	0.643	0.616	0.618
Adjusted R^2^	0.607	0.612	0.561	0.563	0.622	0.624	0.595	0.596

a *p* < 0.1.

b *p* < 0.05.

c *p* < 0.01.

d Other independent variables represent the county’s PM_2.5_ status with respect to the 2006 and 2012 standards and the national gas/coal unit price. For each dependent variable, the first column shows the results of eq 2, and the second column shows the results of eq 4. Unit-level fixed effects are included.

Figure 1 shows no significant difference in emissions reduction for the 99th percentile daily maximum emissions hours compared with 50th percentile emissions for either policy indicator, meaning hypothesis #2 is not supported. The policy coefficient associates the county designation date with a 31.6% drop (95% CI [−0.381, −0.247]) in the 99th percentile emissions metric and a 34.4% drop (95% CI [−0.424, −0.253]) in the 50th percentile emissions metric. Meanwhile, each post-2010 monitor addition is associated with a 12.4% drop (95% CI [−0.188, −0.054]) in the 99th percentile emissions measure and a 14.4% drop (95% CI [−0.228, −0.050]) in the 50th percentile emissions measure. In both cases, the difference is not found to be significant at the p = 0.05 level when comparing z-scores using the method in Paternoster et al.^32^

The lack of a statistically significant difference between reductions at the 50th and 99th percentile suggests that operators implemented SO_2_ control strategies that abated overall SO_2_ emissions but did not specifically target peak emissions. This could be explained by the fact that, while the EPA suggested that states prioritized curbing peak emissions hours,^10^ it may not have offered practical guidance on what policies might incentivize peak reductions separately from average reductions. Instead, rather than requiring states to implement SO_2_ emissions standards with the same averaging time as the NAAQS, the EPA recommends that states adjust existing limits by a multiplier to account for variability at the 99th percentile of emissions. Ultimately, it is more difficult to curb peak EGU emissions than average emissions, as it would require controlling the variability in fuel composition, in combustion, and in the operation of control technologies. The lack of EPA guidance for states on how to adjust their regulatory strategies to align with the hourly standard may explain why the results do not reflect peak emission reductions beyond average reductions that might have been achieved by maintaining the existing annual and daily standards but reducing the permitted concentration level.

Overall, these results are consistent with hypothesis #1 that plants reduced SO_2_ emissions in response to the implementation of the 2010 SO_2_ NAAQS but do not support hypothesis #2 that peak hourly emissions decreased more than median hourly emissions. This suggests that the change in the metric did serve to increase the stringency of the existing standard but may not have shifted the distribution of emissions away from peak hours.

### 3.2 Variation in Emissions by Implementation Round

The second column for each emissions metric in Table 2 shows that the 2010 SO_2_ NAAQS implementation indicator was associated with statistically significant SO_2_ reductions on average for round 2 and round 3 EGUs at the *p* = 0.05 level across all emissions metrics, but round 1 EGU SO_2_ emissions reductions were only statistically significant for the 99th percentile daily maximum hours. Furthermore, Table 3 shows that the SO_2_ ambient monitor addition coefficients for round 2 EGUs were not significant for any SO_2_ emissions metric. Figure 2 presents the percentage SO_2_ emissions reductions by round presented in Tables 2 and 3 and shows that, for both policy metrics, the estimated reduction was not greater on average for EGUs in rounds 1 and 2 relative to round 3, contrary to hypothesis #3. In fact, the policy implementation indicator was associated with greater emissions reductions for round 3 EGUs relative to rounds 1 and 2.

**Figure 2:**
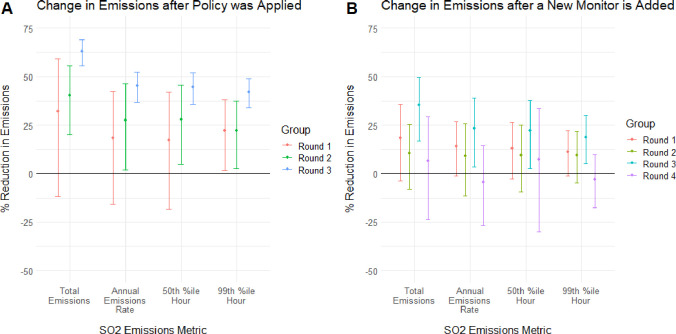
Policy-associated emissions reductions by implementation round. Percentage reductions in each of the four measures of emissions correspond to the coefficients on rounds estimated using eq 3 (plot A) and eq 4 (plot B). Error bars are calculated using robust standard errors clustered at the power plant level. Regressions only included EGUs in the balanced data set (i.e., in operation for all years between 2001 and 2019).

Panel A in Figure 2 shows the emissions changes for each round on and after the date that each county was designated under the 2010 SO_2_ NAAQS. Conducting one-sided *t* tests using the method from Paternoster et al.,^32^ the difference between the round 1 and 3 coefficients was significant at the p = 0.05 level for all measures of emissions reduction, and the difference between the round 2 and 3 coefficients was significant for annual emissions and the 99th percentile of daily emissions hours. Otherwise, no other difference between round coefficients was significant. Plot B in Figure 2 shows the emissions changes for each round following the installation of a new ambient SO_2_ monitor nearby. However, for the new monitor count independent variable, the estimated change was only statistically significantly greater for round 3 than round 2 for annual emissions at the p = 0.05 level, and there were no statistically significant differences between round 1 and 3 emissions changes at the p = 0.05 level. Therefore, there is not enough evidence to support hypothesis #3.

The results suggest that despite their higher level of priority according to the EPA,^12^ round 2 EGUs did not reduce emissions more, and may have reduced them less, than round 3 EGUs following the policy change. Furthermore, there is some evidence that round 1 EGUs reduced emissions less than units in round 3, though the relatively low number of plants in round 1 (45 EGUs) relative to round 3 (268 EGUs) means that the magnitude of the difference cannot be determined. This suggests the 2010 SO_2_ NAAQS change may not have effectively targeted the EGUs most likely to cause violations. This could be because these EGUs have technical or organizational characteristics that make reducing emissions more difficult. This is supported by the timings of new control technology installations, which show that a relatively small percentage of round 2 plants installed new control technologies after the policy implementation compared with round 3 plants.

One alternative explanation for the difference in average SO_2_ reductions across rounds may be differences in the SO_2_ control technology deployment. Panel A in Figure 3 shows the overall proportion of EGUs in each policy round with SO_2_ control technologies installed each year. While a similar proportion of EGUs in rounds 1, 3, and 4 had SO_2_ controls installed in 2001, by 2019 round 3 SO_2_ control deployment had increased above round 1 deployment by 11 percentage points and above round 4 deployment by 28 percentage points on a unit basis. This may explain why round 1 and 4 EGUs were less responsive to 2010 SO_2_ NAAQS implementation.

**Figure 3:**
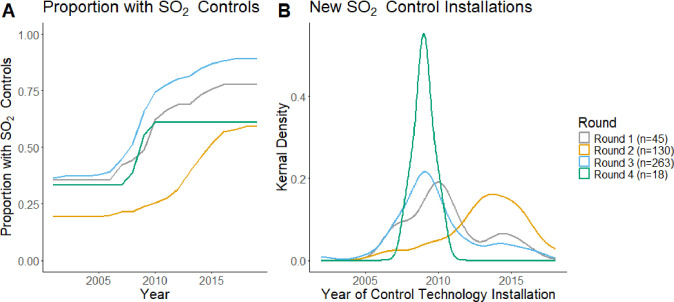
Proportion of EGUs with control technology and distribution of installations by year and policy implementation round, calculated for coal EGUs in the balanced panel.

Panel A in Figure 3 also shows that round 2 EGUs—which were more likely to be at risk of causing a NAAQS violation, consistently had the lowest rates of SO_2_ control deployment. The lower rate of SO_2_ control installation among round 2 EGUs in 2001 is consistent with the EPA’s selection criteria for round 2 counties, since EGUs without SO_2_ controls installed would be more likely to meet the annual SO_2_ emissions threshold that would trigger the earlier designation date. Panel A shows that despite the lack of a statistically significant effect of the 2010 SO_2_ NAAQS policy on SO_2_ emissions of round 2 EGUs, the rate of SO_2_ control technology deployment for round 2 EGUs more than tripled from 2001 to 2019. This may indicate that while rates of SO_2_ emissions control deployment increased for EGUs in all rounds, for round 3 EGUs, emissions reductions were most likely to be associated with the 2010 SO_2_ NAAQS implementation. Conversely, emission reductions by round 2 EGUs may have been less strongly associated with 2010 SO_2_ NAAQS promulgation and may instead have occurred in response to other policies.

The timing of new SO_2_ control technology installations is consistent with the possibility that EGUs in round 2 may have been more responsive to environmental policies other than the 2010 SO_2_ NAAQS. In Figure 3, the increase in penetration of SO_2_ control technology among round 2 EGUs in Panel A appears to begin at a later date than the deployments among EGUs in other rounds. The difference in timing of SO_2_ control installations between rounds is visualized more directly in Panel B. For EGUs in round 2, new control installations stopped increasing year-on-year around 2013–2015, compared with around 2010 for round 3 plants. The relatively late deployment of SO_2_ controls of round 2 EGUs compared with EGUs in other rounds may explain the lower proportional emissions reductions achieved by these EGUs in response to the SO_2_ NAAQS revision. It may also indicate that these installations were incentivized by a set of policies different from those seen for EGUs in other rounds.

### 3.3 Policy Implications

Our analysis finds that the 2010 SO_2_ NAAQS revision was associated with lower SO_2_ emissions at coal-fired power plants in terms of average annual emissions, emissions rate, median hourly emissions, and 99th percentile hourly emissions. This reduction in SO_2_ emissions is associated with both the date of initial designation under the new standard as well as the addition of nearby ambient SO_2_ monitors that determined compliance with the 2010 SO_2_ standard. Our result is generally consistent with the result in Greenstone (2004)^19^ which showed modest reductions in ambient SO_2_ concentrations following implementation of the 1972 SO_2_ NAAQS, though unlike in Greenstone, the effect found here for SO_2_ emissions is statistically significant. One explanation for this finding could be the use of SO_2_ emissions rather than SO_2_ concentrations as the dependent variable, since concentrations are determined by many meteorological and other nonanthropogenic influences that may dampen the relationship with policy.

Our results show that the 2010 SO_2_ revision may have provided some protection against acute exposure, since average reductions in peak SO_2_ emissions across plants were statistically significant. However, the proportional reduction in peak emissions was not statistically significantly different from the reductions in the median or average emissions. This result is consistent with emissions reductions in response to the increased stringency in the ambient SO_2_ limit but not with a change in the targeting of the standard. This could be due to how the SO_2_ NAAQS was enforced at the state level. The EPA’s SIP recommendations offer states flexibility to set emissions limits with averaging times of up to 30 days in order to achieve or maintain compliance.^10^ Such emissions limits must be set to ensure that the 99th percentile of predicted hourly emissions would be below the level that would raise concentrations above the NAAQS limit. However, the EPA guidance acknowledges that future distributions of plant SO_2_ emissions can be difficult to predict, particularly for peaker plants or plants with SO_2_ scrubbers that are not required to operate them continuously. One explanation for the similar proportional reduction in median and peak SO_2_ emissions might be that on average, states pursued regulatory strategies that would achieve hourly attainment by requiring even greater reductions in average emissions rather than by shifting the emissions distribution.

The purpose of revising the SO_2_ NAAQS to an hourly standard was to mitigate acute SO_2_ exposures. The 2010 revision responded to experimental evidence showing that acute exposures to SO_2_ could trigger respiratory symptoms in asthmatics while repeated exposure to low levels of ambient SO_2_ had minimal effect.^33,34^ These experimental findings are supported by subsequent observational studies^35^ and meta-analyses^1^ highlighting the importance of limiting exposures to high ambient SO_2_ concentrations to protect public health. However, to achieve the peak emission reductions that we observe, it may have been sufficient for the EPA to maintain the annual and daily primary standards but reduce their concentration limits. This would have allowed state regulatory bodies to forego the substantial resource investments necessary to judge attainment with the new standard and reduced ambiguity around how the new rule would be enforced. Alternatively, the hourly concentration standard may have more effectively targeted peak emissions if the EPA had provided further guidance on how states might enforce emission standards with shorter averaging times at the plant level. For example, the EPA guidance might have placed a greater emphasis on state regulations that control peak emissions variability by mandating SO_2_ scrubber operation or capping generation during hours when SO_2_ NAAQS violations are most likely. Such guidance would allow states even greater flexibility in how states regulate plant behavior to achieve attainment and may have resulted in more efficient regulatory outcomes.

When comparing the effects of the SO_2_ NAAQS revision between EGUs in different implementation rounds, we find that the greatest reductions in SO_2_ were made by EGUs that already had low absolute SO_2_ emissions. These EGUs were also in counties that were not otherwise required to deploy SO_2_ ambient monitors sufficient to judge compliance with the 2010 SO_2_ NAAQS, suggesting that the counties were not previously considered at high risk of violating the existing standard. Meanwhile, ambient SO_2_ monitor additions did not have statistically significant effects on EGUs in round 2 which may have been more likely to directly contribute to NAAQS violations. This result may indicate that the EGUs most likely to contribute to acute SO_2_ exposure are less responsive to the incentives created through state implementation of the NAAQS. Alternatively, these EGUs may have been more likely to be targeted by other emissions control policies such as MATS or regional SO_2_ trading programs, and their emissions reductions may have been more in response to these programs and less correlated with the SO_2_ NAAQS revision.

Overall, this analysis demonstrates how the effects of a policy depend not only on the text of the regulation but also on the details of its implementation. Analyzing the effects of regulations ex-post provides insight into firm-level responses and reveals differences between a policy on paper and in practice. Quantifying the effects of air quality policy on plant emissions can be used to inform future analyses of the expected benefits of such policies on public health. Understanding the gaps between policy intent and outcomes can help policymakers in areas where additional guidance may be necessary to help states achieve the desired outcomes. Finally, many policy evaluations assume that policy is implemented uniformly and do not use sufficiently disaggregated data to examine impacts on the outcome targeted by the standard. This study shows the potential value of using increasingly detailed, unit-level data to assess compliance with changes in policy targeting, as well as stringency.

## Supplementary Material



## Notes

1) Descriptive data on coal-fired EGUs; 2) policy treatment effects over time; 3) robustness checks that estimate results when 3.1) using an indicator variable for monitor addition, 3.2) decreasing the radius of monitor additions, 3.3) using untransformed emissions measures, 3.4) controlling for fuel price by unit, and 3.5) controlling for annual fixed effects; 4) estimating implementation round based on EGU characteristics; and 5) discussion of data on EGU SIP violations (PDF)
