# Supplementary material for: Changes in the Temporal Targeting of the U.S. National Ambient Air Quality Standards (NAAQS) for SO2 Reduce Average and Peak Emissions from Coal Power Plants
Source: Environ Sci Technol. 2025 Jun 18;59(25):12437–46. doi: 10.1021/acs.est.4c10718 (PMC12224305; doi:10.1021/acs.est.4c10718)
Supplement: Supplementary file 1 [file es4c10718_si_001.pdf]

# Supplemental Information: Changes in the temporal targeting of the U.S. National Ambient Air Quality Standards (NAAQS) for $SO_2$ reduce average and peak emissions from coal power plants

Joanna H. Slusarewicz[a,*], Valerie J. Karplus[a,b]

April 2025

Summary: 33 pages, 9 figures, 13 tables

[a]Department of Engineering and Public Policy, Carnegie Mellon University, 5000 Forbes Ave, Pittsburgh, PA 15213, United States

[b]Wilton E. Scott Institute for Energy Innovation, Carnegie Mellon University

[*]Corresponding author. Email: jslusare@andrew.cmu.edu.

# 1 Full Dataset Description

Data processing began with the facility data provided by the Air Markets Program regarding all power plants regulated under EPA emissions trading programs. Facility data consists of the EGUs' name, location, ownership, fuel types, control technologies, and other operational features for each year, and included 4795 unique EGUs. Since this analysis utilized emissions data from 2001-2019, the dataset was limited to facilities that operated at any point within this time period, of which there were 4442 unique EGUs. This list of EGUs was then further narrowed to only include EGUs that used coal as their primary fuel in a given year, leaving 1176 EGUs in the dataset that used primarily coal at any point between 2001 and 2019. This limited dataset of annual facility attributes was then merged with annual emissions metrics from CEMS reporting provided by the Air Markets program, with state-level natural gas prices over national coal prices by year, and with county-level $PM_{2.5}$ classifications.

The main text only considered EGUs that operated continuously from 2001-2019, which resulted in 456 EGUs in the balanced dataset. This was done because it was not possible to run regressions on the logarithm of EGU $SO_2$ emissions if the $SO_2$ emissions were 0 for a given year. If observations with 0 emissions had been dropped, the regressions would not have captured emissions reductions due to the EGU closure. Therefore, including EGUs that closed down or switched to natural gas from 2001-2019 would not capture their full response to the policy, making the regression results less interpretable.

The rest of this appendix summarizes EGU emissions behaviors for the full dataset, complementing the summary of the balanced dataset in the main text. Figure S1 shows the distribution of emissions quantities and rates by round for all EGUs from 2010 to 2012. The emissions discrepancies between rounds were similar to the balanced dataset, with round 1 and 2 EGUs having greater emissions both annually and for the $99^{th}$ and $50^{th}$ percentiles of daily maximum hours. The main difference between the balanced and unbalanced emissions data is the spread of observations, where the unbalanced data showed a greater spread of emissions amounts, particularly at the low end. This is likely because the additional EGUs in the unbalanced dataset were largely those which stopped operating in later years, and therefore may have had relatively lower generation

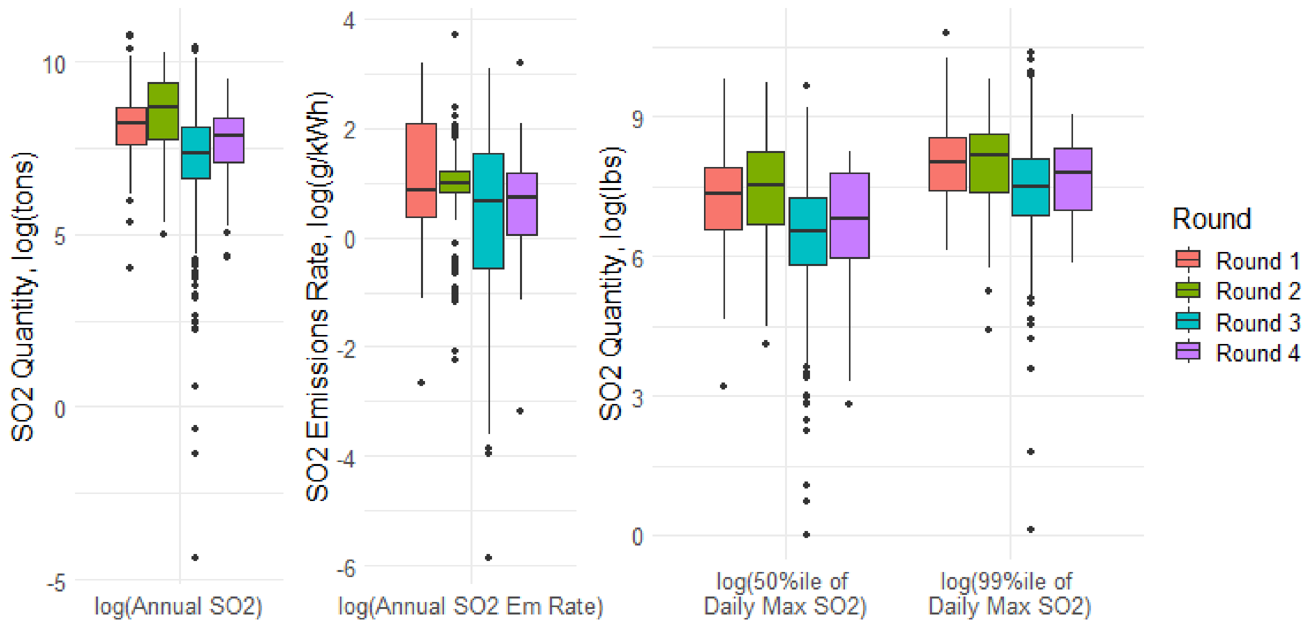


Figure S1: Distributions of EGU average $SO_2$ emissions from 2010 to 2012 by round and emissions metric. Includes all coal plants from the unbalanced dataset.

preceding closure.

Figure S2 shows that average generation for operating coal-fired EGUs each year was indeed lower for plants in the unbalanced dataset preceding 2010 relative to the average for EGUs that remained open through 2019. Though there does not appear to be a decline in the average generation among all coal EGUs, this is because Figure S2 shows the average generation among *operating* EGUs each year. Figure S3 shows that total generation did in fact decline as more coal plants closed in the 2010s.

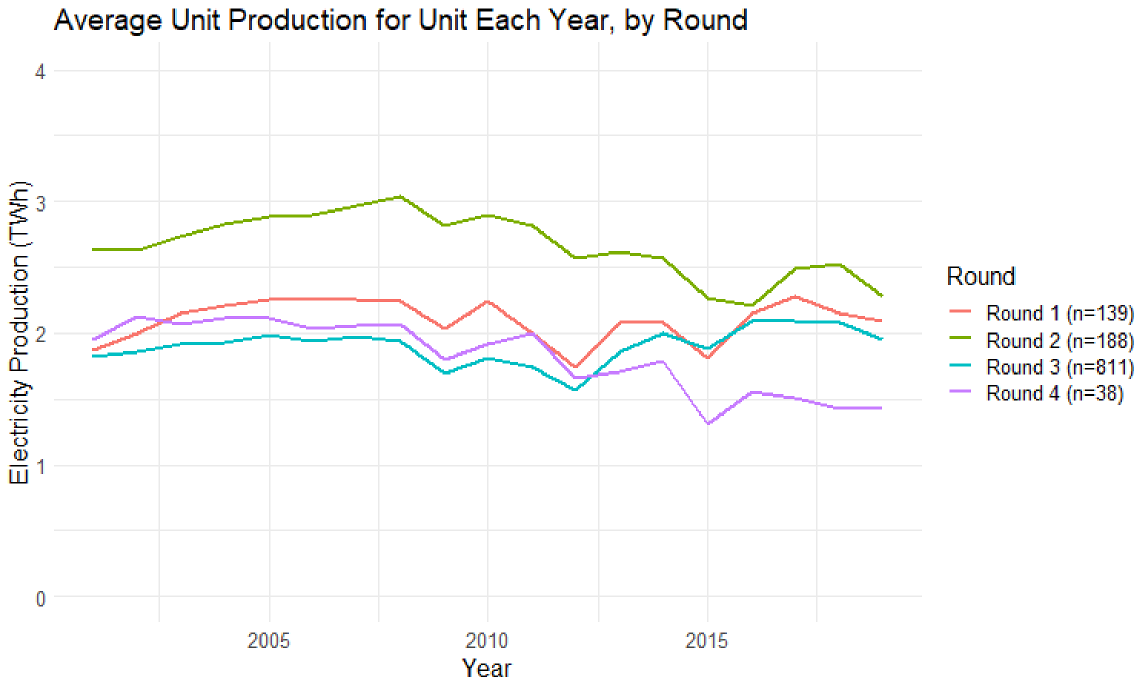


Figure S2: Average electricity production among EGUs in each round, per year.

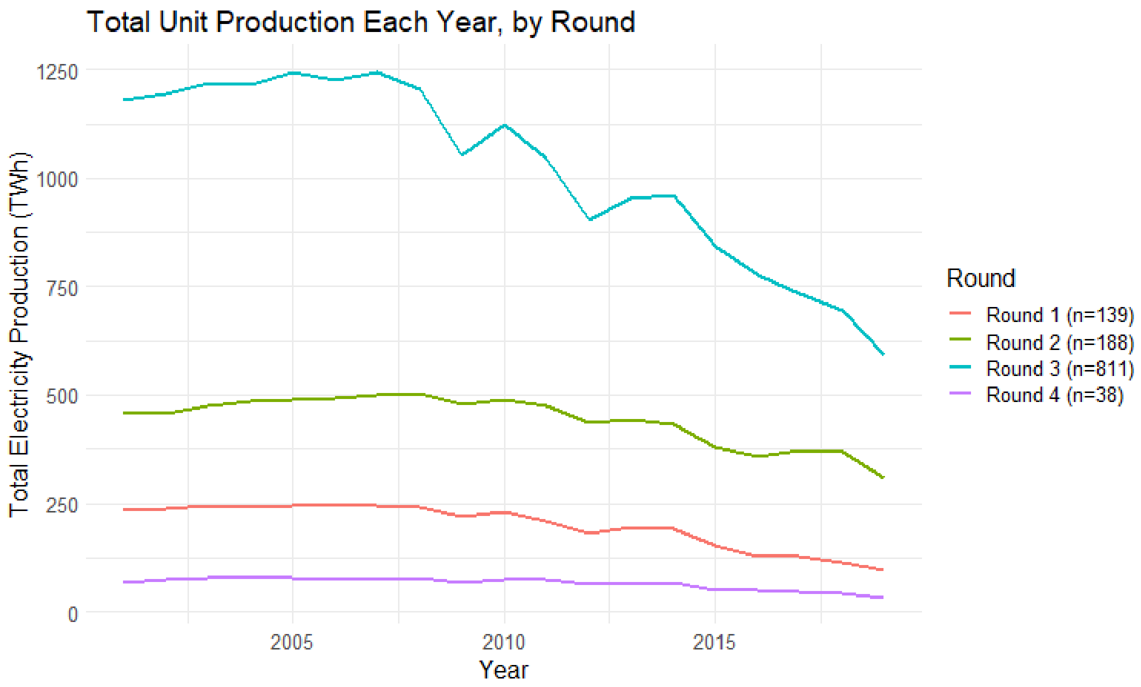


Figure S3: Total electricity production from all coal EGUs in each round, per year.

## 2 Treatment Effects

The follow section describes pre- and post-treatment effects of policy indicators for the balanced dataset. In this equation, note that $\tau$ represents the number of years between the initial 2010 $SO_2$ NAAQS designation date of the county and the observation year. Therefore, coefficients $\beta_\tau$ when $\tau$ is less than 0 represent the anticipatory effects of the policy and when $\tau$ is greater than or equal to 0 they represent the post-treatment effects.

$$ln(\text{Unit } SO_2)_{i,t} = \alpha_0 + \gamma_i + \beta_P P_t + \beta_{\text{PM06}}\text{PM06}_{c,t} + \beta_{\text{PM12}}\text{PM12}_{c,t} + \sum_{\tau=-q}^{\tau=m} \delta_\tau + \epsilon_{i,t} \quad (1)$$

Figure S4 shows the results from running the regression in equation 1 on the log of EGU $SO_2$ emissions outcomes for EGUs in the balanced dataset.

Further, while regressions using total monitor additions per year cannot be plotted using simple leads and lags, we can also consider the pre- and post-treatment effects of the first post-2010 ambient monitor being added near EGUs. Figure S5 shows the results of running equation 1 where $\tau$ instead represents the number of years before or after the first post-2010 monitor was added near an EGU. Note that while there is a downward slope in log(emissions) for all emissions variables moving forward in time, the slope of the reduction seems to get steeper when the lag is 0.

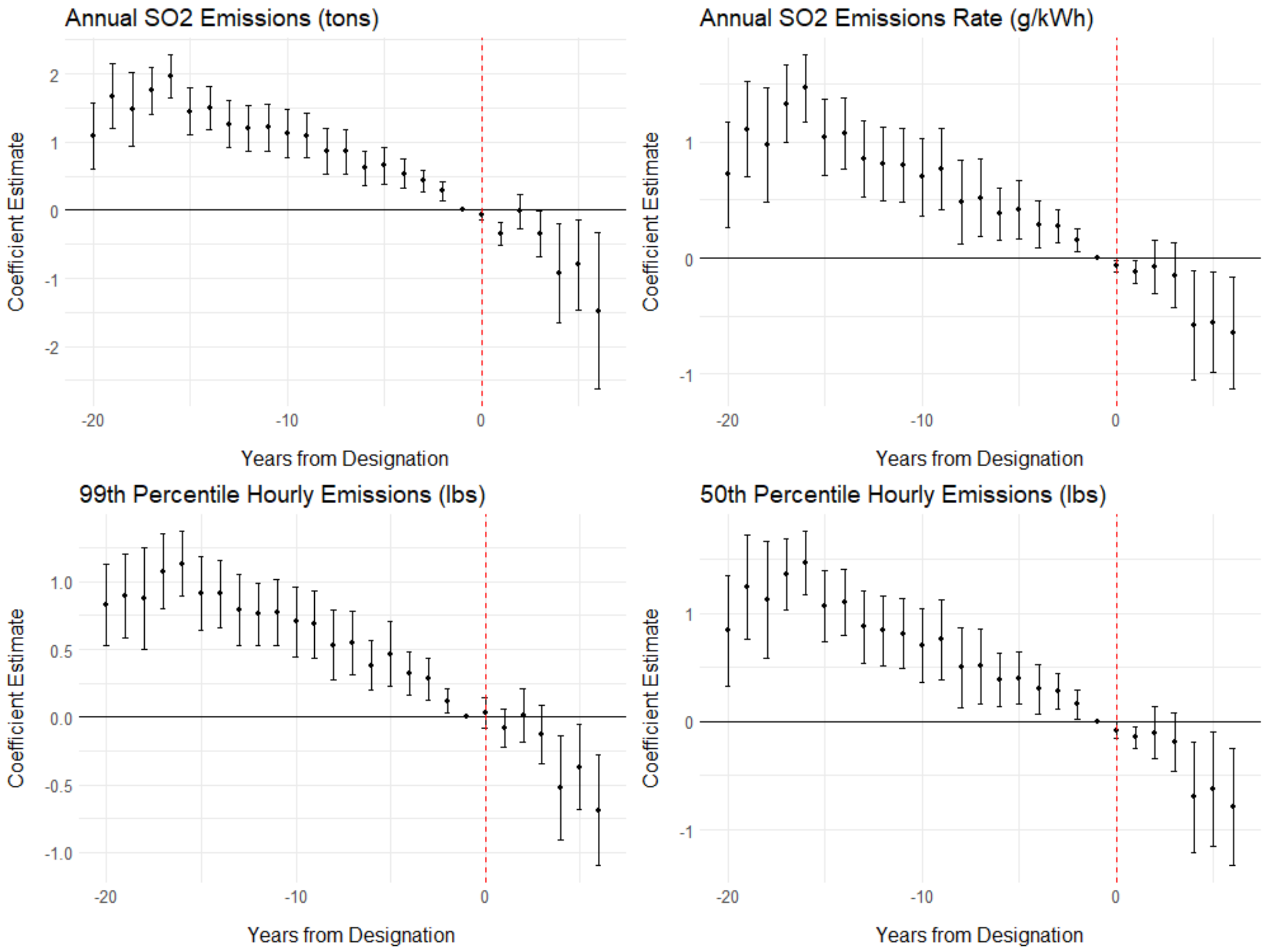


Figure S4: Anticipatory and post-treatment effects of 2010 $SO_2$ NAAQS designation on EGUs' $SO_2$ emissions outcomes.

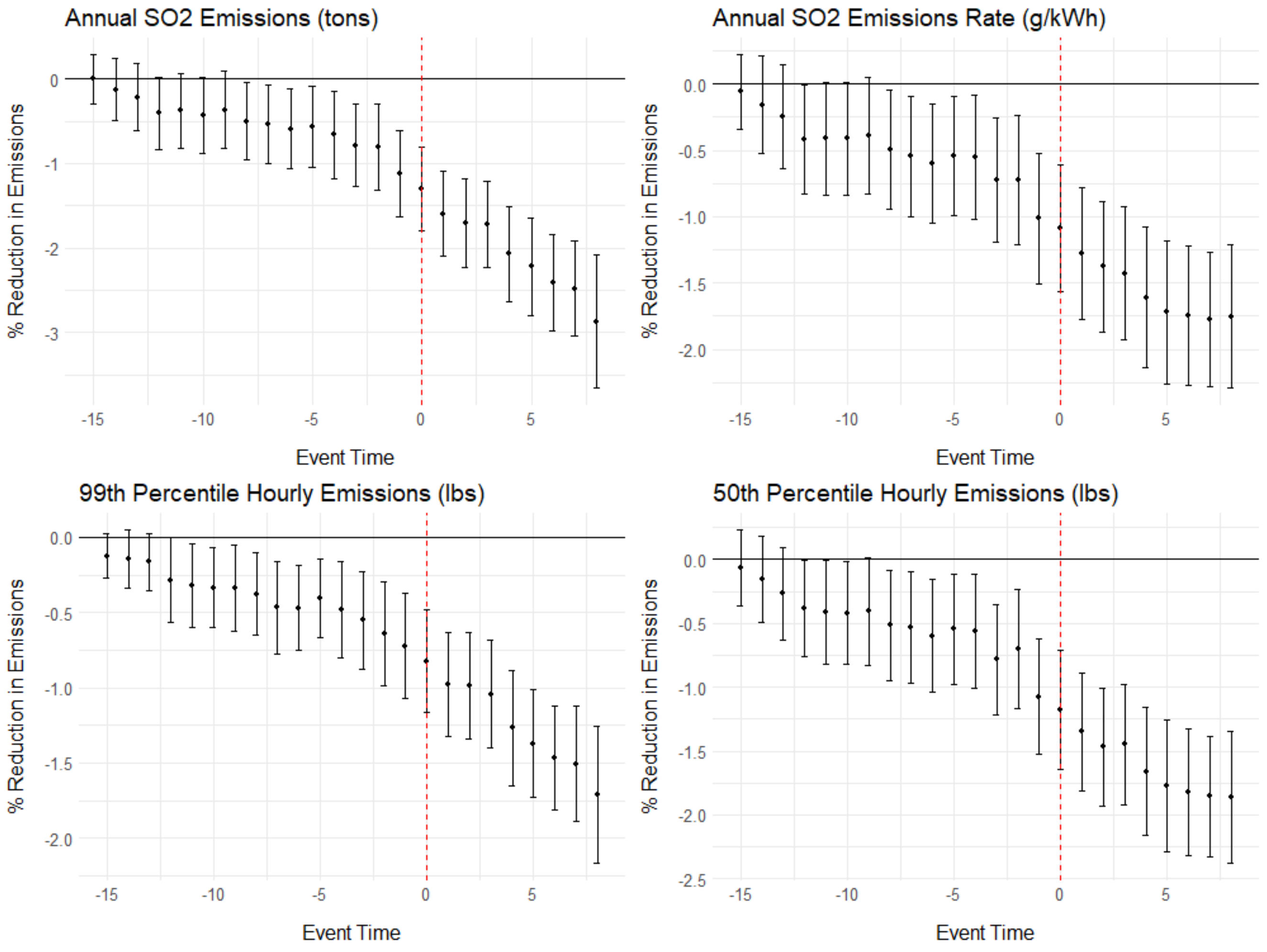


Figure S5: Anticipatory and post-treatment effects of the first post-2010 addition of a new monitor near EGUs.

# 3 Regression Robustness

## 3.1 Regression with Post-2010 Monitor Indicator

The regression results shown in Table ?? show the results of running regressions 2 and 4 on the balanced dataset. However, instead of representing the number of post-2010 monitor additions, $A_{i,t}$ represents an indicator equal to 1 when a new monitor had been installed to assess 2010 $SO_2$ NAAQS compliance before or during year $t$ within 50 km of EGU $i$. The results show that after the first new monitor is added near an EGU, there are associated decreases in all measures of emissions outcomes. Further, the decreases associated with the first new monitor addition are greater than the per-monitor decrease found in Table 3 in the main paper, suggesting that the emission-dampening effect grows weaker as more new monitors are added near the same EGU.

Table S1: Resulting coefficient estimates after regressing each of the four unit emissions measures on an indicator equal to 1 in and after the year any new ambient $SO_2$ monitor was added within 50 km of a unit since 2010. Other independent variables represent the county's $PM_{2.5}$ status with respect to the 2006 and 2012 standards and the national gas/coal unit price. For each dependent variable, the first column shows the results of equation (2) and the second column shows the results of equation (4).

| | *Dependent variable:* | | | | | | | |
|---|---|---|---|---|---|---|---|---|
| | log(Annual SO2 (tons)) | | log(Annual SO2 Rate (g/kWh)) | | log(99%ile SO2 Hour (lbs)) | | log(50%ile SO2 hr (lbs)) | |
| Post-2010 50 km Monitor Indicator | −0.701*** | | −0.470*** | | −0.336*** | | −0.492*** | |
| | (0.136) | | (0.127) | | (0.087) | | (0.129) | |
| Post-2010 50 km Monitor Indicator (R1) | | −0.687** | | −0.465* | | −0.337* | | −0.467* |
| | | (0.346) | | (0.248) | | (0.204) | | (0.239) |
| Post-2010 50 km Monitor Indicator (R2) | | −0.192 | | −0.063 | | −0.056 | | −0.099 |
| | | (0.170) | | (0.182) | | (0.131) | | (0.174) |
| Post-2010 50 km Monitor Indicator (R3) | | −1.113*** | | −0.816*** | | −0.575*** | | −0.787*** |
| | | (0.188) | | (0.181) | | (0.119) | | (0.189) |
| Post-2010 50 km Monitor Indicator (R4) | | −0.262 | | 0.005 | | 0.009 | | −0.334 |
| | | (0.351) | | (0.242) | | (0.127) | | (0.481) |
| PM2.5 Nonattainment (2006) | −0.036 | −0.013 | −0.037 | −0.016 | 0.176 | 0.190* | −0.169 | −0.154 |
| | (0.217) | (0.209) | (0.167) | (0.166) | (0.118) | (0.113) | (0.178) | (0.175) |
| PM2.5 Nonattainment (2012) | −0.381*** | −0.397** | −0.028 | −0.038 | −0.153 | −0.159 | 0.116 | 0.097 |
| | (0.116) | (0.192) | (0.203) | (0.232) | (0.105) | (0.141) | (0.095) | (0.127) |
| Gas/Coal Price | 0.476*** | 0.474*** | 0.360*** | 0.358*** | 0.261*** | 0.260*** | 0.370*** | 0.368*** |
| | (0.035) | (0.035) | (0.032) | (0.032) | (0.021) | (0.022) | (0.032) | (0.033) |
| Constant | 7.134*** | 7.140*** | −0.665*** | −0.659*** | 7.778*** | 7.782*** | 6.567*** | 6.572*** |
| | (0.108) | (0.109) | (0.099) | (0.099) | (0.066) | (0.066) | (0.100) | (0.100) |
| Unit FE | Yes | Yes | Yes | Yes | Yes | Yes | Yes | Yes |
| Observations | 8,778 | 8,778 | 8,778 | 8,778 | 8,778 | 8,778 | 8,772 | 8,772 |
| $R^2$ | 0.634 | 0.641 | 0.588 | 0.595 | 0.643 | 0.648 | 0.621 | 0.626 |
| Adjusted $R^2$ | 0.614 | 0.621 | 0.565 | 0.572 | 0.623 | 0.628 | 0.600 | 0.605 |

*Note:* $^{*}p<0.1$; $^{**}p<0.05$; $^{***}p<0.01$

## 3.2 Regressions on Monitor Additions within an Alternative Radius

The main analysis uses monitor additions within 50 km of an EGU as a measure of 2010 $SO_2$ NAAQS implementation, which corresponds with "urban-scale" monitoring according to the EPA. This radius corresponds to the largest "appropriate spatial scale" for 5-minute $SO_2$ ambient monitors to be used for characterizing local air quality towards $SO_2$ NAAQS attainment decisions.[1] An ambient $SO_2$ monitor added within 50 km of a power plant may indicate that the power plant is directly subject to additional scrutiny under the 2010 $SO_2$ NAAQS update. Alternatively, a monitor added within 50 km suggests that a plant is located near an area where the plant's emissions could impact attainment status.

This section compares the effect of ambient $SO_2$ monitor additions within 50 km of coal EGUs to the effect of monitor additions within 4 km. According to the EPA's Network Design Criteria, 4 km corresponds to the coverage radius of monitors that characterize "neighborhood scale" air quality.[1] One stated use case of these monitors is to estimate the contribution of $SO_2$ emissions from a stationary source to local air quality.

Table S2 shows the number of EGUs in each round and the number of EGUs that had a monitor installed after 2010 within 50 km and within 4 km at any time after the 2010 $SO_2$ NAAQS revision was promulgated. For every group except round 2, there were substantially fewer EGUs that had $SO_2$ monitor additions within a 4 km radius relative to a 50 km radius, particularly for EGUs in the third implementation round. By comparison, over 60% of round 2 EGUs that had an $SO_2$ monitor added within 50 km of the EGU also had a monitor added at the neighborhood scale. This is consistent with the fact that counties designated in round 2 of the 2010 $SO_2$ NAAQS revision were usually placed in this group due to the presence of major $SO_2$ sources. Placement may indicate that these sources were indeed being regulated more stringently under state law to facilitate NAAQS compliance.

The regression results presented in Table S3 show the effect of each additional monitor installed within 4 km of an EGU on that EGU's emissions. Columns 1, 3, 5 and 7 show the average effect across EGUs of a monitor added within 4 km of an EGU, and the results are visualized in Figure S6. An additional monitor added within 4 km of a plant was associated with a reduction in all

Table S2: Number of units that had monitors added 4 km away and 50 km away after 2010, by 2010 SO2 NAAQS implementation round.

| Round | Total Units | Added 50km monitor | Added 4km monitor |
|---|---|---|---|
| Round 1 | 45 | 28 | 8 |
| Round 2 | 130 | 69 | 42 |
| Round 3 | 263 | 103 | 14 |
| Round 4 | 18 | 17 | 8 |

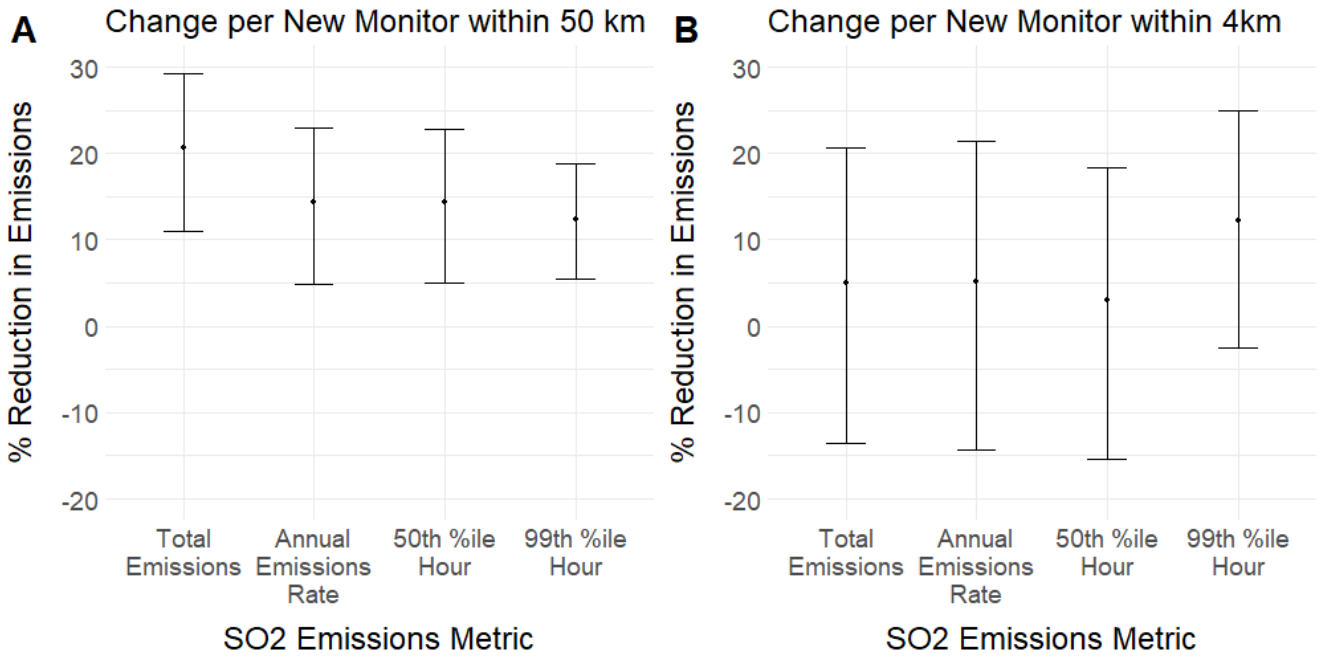


Figure S6: Estimated effect of $SO_2$ ambient monitor additions on proportional $SO_2$ emissions reductions by coal EGUs. Panel A shows the estimated effect of each additional ambient monitor installation within 50 km of an EGU and Panel B shows the effect of monitors added within 4 km.

metrics of $SO_2$ emissions just as with monitors within 50 km, although reductions were more uncertain and no longer statistically significant, likely due to the smaller sample size.

Columns 2, 4, 6 and 8 in Table S3 show the effect of monitor additions within 4 km of coal EGUs by implementation round, and Figure S7 compares the magnitudes of the associated $SO_2$ emissions changes with the effects of monitors added within 50 km of EGUs. The by-round estimates for 4 km monitors reflect similarly wide uncertainty bounds, although round 3 EGUs do appear to broadly perform better as with 50 km monitors.

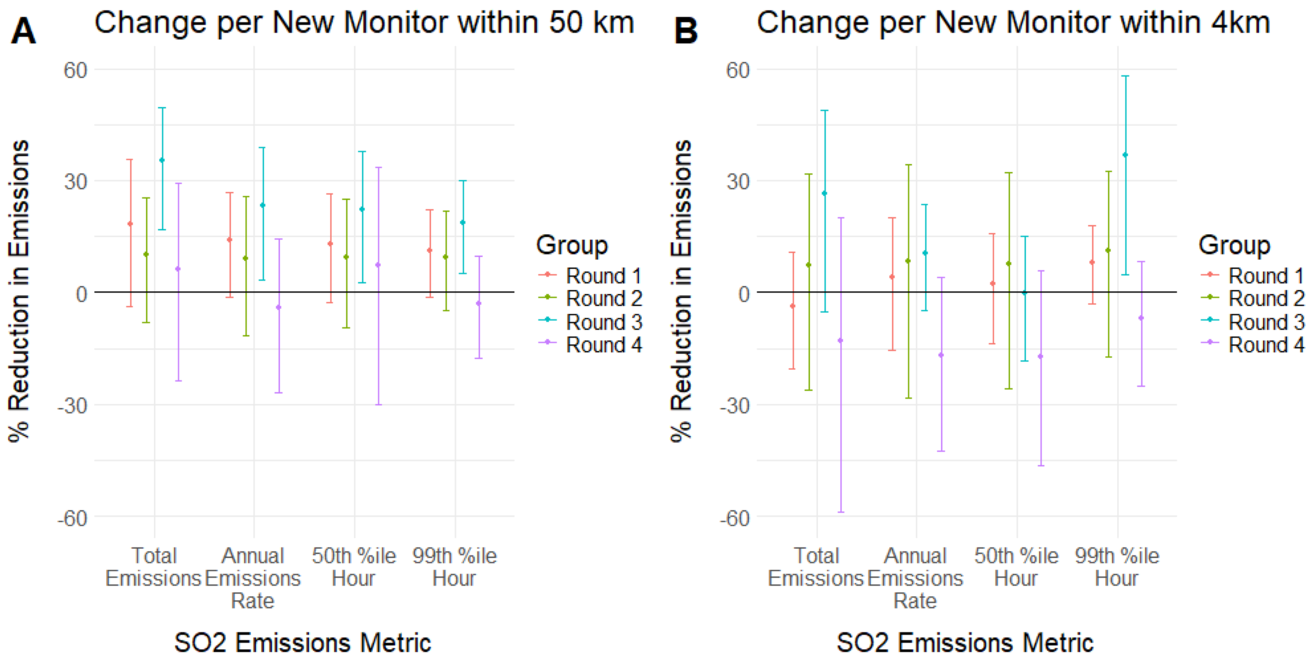


Figure S7: Estimated effect of $SO_2$ monitor additions on proportional $SO_2$ emissions reductions at coal-fired EGUs by 2010 $SO_2$ NAAQS implementation round. Panel A shows the estimated effect of each additional ambient monitor installation within 50 km of an EGU and Panel B shows the effect of monitors added within 4 km.

Table S3: Resulting coefficient estimates after regressing each of the four unit emissions measures on new ambient $SO_2$ monitors added within 4 km of the unit since 2010. Other independent variables represent the county's $PM_{2.5}$ status with respect to the 2006 and 2012 standards and the national gas/coal unit price. For each dependent variable, the first column shows the results of equation (2) and the second column shows the results of equation (4).

| | *Dependent variable:* | | | | | | | |
|---|---|---|---|---|---|---|---|---|
| | log(Annual SO2 (tons)) | | log(Annual SO2 Rate (g/kWh)) | | log(99%ile SO2 Hour (lbs)) | | log(50%ile SO2 hr (lbs)) | |
| Post-2010 4km Monitor Count | −0.052 | | −0.053 | | −0.131 | | −0.030 | |
| | (0.091) | | (0.095) | | (0.080) | | (0.089) | |
| Post-2010 4km Monitor Count (Round 1) | | 0.037 | | −0.040 | | −0.083 | | −0.021 |
| | | (0.076) | | (0.094) | | (0.058) | | (0.076) |
| Post-2010 4km Monitor Count (Round 2) | | −0.074 | | −0.086 | | −0.116 | | −0.078 |
| | | (0.157) | | (0.171) | | (0.141) | | (0.157) |
| Post-2010 4km Monitor Count (Round 3) | | −0.308* | | −0.112 | | −0.457** | | 0.002 |
| | | (0.184) | | (0.081) | | (0.209) | | (0.085) |
| Post-2010 4km Monitor Count (Round 4) | | 0.121 | | 0.156 | | 0.069 | | 0.161 |
| | | (0.175) | | (0.101) | | (0.080) | | (0.113) |
| PM2.5 Nonattainment (2006) | 0.041 | 0.039 | 0.014 | 0.012 | 0.204* | 0.203* | −0.114 | −0.116 |
| | (0.224) | (0.224) | (0.165) | (0.165) | (0.119) | (0.119) | (0.178) | (0.178) |
| PM2.5 Nonattainment (2012) | −0.596* | −0.596* | −0.173 | −0.173 | −0.264 | −0.264 | −0.035 | −0.035 |
| | (0.334) | (0.333) | (0.365) | (0.364) | (0.215) | (0.214) | (0.152) | (0.152) |
| Gas/Coal Price | 0.545*** | 0.545*** | 0.405*** | 0.405*** | 0.289*** | 0.289*** | 0.418*** | 0.418*** |
| | (0.036) | (0.036) | (0.031) | (0.031) | (0.021) | (0.021) | (0.032) | (0.032) |
| Constant | 6.922*** | 6.923*** | −0.805*** | −0.804*** | 7.690*** | 7.690*** | 6.419*** | 6.419*** |
| | (0.110) | (0.110) | (0.097) | (0.097) | (0.066) | (0.066) | (0.098) | (0.098) |
| Unit FE | Yes | Yes | Yes | Yes | Yes | Yes | Yes | Yes |
| Observations | 8,778 | 8,778 | 8,778 | 8,778 | 8,778 | 8,778 | 8,772 | 8,772 |
| $R^2$ | 0.618 | 0.619 | 0.577 | 0.578 | 0.637 | 0.638 | 0.611 | 0.611 |
| Adjusted $R^2$ | 0.597 | 0.597 | 0.554 | 0.554 | 0.617 | 0.618 | 0.589 | 0.589 |

*Note:* *p<0.1; **p<0.05; ***p<0.01

## 3.3 Regressions with Unlogged $SO_2$ Variables

The logarithms were taken for $SO_2$ emissions outcomes in the functional forms chosen for the main regressions. This is because, for all equations and emissions outcomes, the lambda values resulting from the box-cox transformation method were close to zero, indicating that a log-linear regression was appropriate to maintain the normality assumption. Regression results using linear emissions quantities are presented below as a robustness check.

Table S4 shows the results of running the regressions from equations 1 and 3 with unlogged $SO_2$ metrics as the dependent variable rather than the logged values. The resulting coefficients represent the quantity of $SO_2$ emissions reduction associated with the EGUs' counties inclusion under the new $SO_2$ NAAQS standard. In particular, we see that implementing the standard change was associated with a of 2190 tons (95% CI [-3190, -1190]) for annual emissions while $SO_2$ emissions rates decreased by 0.687 g/kWh (95% CI [-0.986, -0.389]).

Table S4 also shows the estimated policy-associated decrease in the $99^{th}$ percentile of daily maximum hour emissions was greater than the decrease in the $50^{th}$ percentile. Specifically, $SO_2$ emissions during daily maximum hours decreased by 945 pounds (95% CI [-1430, -464]) at the $99^{th}$ percentile and by 566 pounds (95% CI [-865, -267]) at the $50^{th}$ percentile. This difference was found to be significant at the p=0.10 level, but not the p=0.05 level.

Meanwhile, Table S5 shows the results of running regressions 2 and 4 on the balanced dataset with raw $SO_2$ quantities rather than the logged values. The results show that in a year after a post-2010 $SO_2$ NAAQS monitor was added near an EGU, that EGU's total annual $SO_2$ emissions could be expected to be 1370 tons less (95% CI [-2090, -646]) than they were in a year before the monitor was added. The associated decrease in $SO_2$ emissions rate in years after a monitor is added was estimated to be 0.362 g/kWh (95% CI [-0.568, -0.157]). There also appears to be a greater decrease in $SO_2$ emissions at the $99^{th}$ percentile of daily maximum hours relative to the $50^{th}$, with $99^{th}$ percentile hourly emissions decreasing by -587 lbs (95% CI [-893, -280]) relative to a decrease of -406 lbs (95% CI [-642, -170]) at the $50^{th}$ percentile. However, this difference was not significant at either the p=0.05 or p=0.10 level.

These results are consistent with hypothesis 1, with policy application and monitor addition

associated with $SO_2$ emissions reductions for all metrics. However, on their own they offer weak support for hypothesis 2, which hypothesized that EGUs' peak hourly emissions would decrease more than their median emissions in response to policy. The $99^{th}$ percentile hourly emissions did decrease more than $50^{th}$ percentile, but this difference was not significant for the monitor addition measure of policy application and was only significant at the p=0.10 level for the county classification measure.

Table S4: Resulting coefficient estimates after regressing each of the four unit emissions measures on a dummy variable indicating whether the 2010 $SO_2$ NAAQS had been implemented in the unit's county. Other independent variables represent the county's $PM_{2.5}$ status with respect to the 2006 and 2012 standards and the national gas/coal unit price. For each dependent variable, the first column shows the results of equation (1) and the second column shows the results of equation (3).

| | *Dependent variable:* | | | | | | | |
|---|---|---|---|---|---|---|---|---|
| | Annual SO2 (tons) | | Annual SO2 Rate (g/kWh) | | 99%ile SO2 Hour (lbs) | | 50%ile SO2 hr (lbs) | |
| Policy Flag | −2,189.731*** | | −0.687*** | | −944.784*** | | −566.200*** | |
| | (509.625) | | (0.152) | | (245.100) | | (152.556) | |
| Policy Flag (R 1) | | −4,152.221 | | −0.941 | | −1,629.626* | | −1,231.476* |
| | | (2,622.013) | | (0.580) | | (942.461) | | (739.805) |
| Policy Flag (R 2) | | −1,681.652** | | −0.442 | | −503.000 | | −278.313 |
| | | (802.272) | | (0.313) | | (431.481) | | (256.797) |
| Policy Flag (R 3) | | −1,884.533*** | | −0.802*** | | −1,064.221*** | | −561.536*** |
| | | (326.358) | | (0.118) | | (236.638) | | (101.741) |
| Policy Flag (R 4) | | | | | | | | |
| PM2.5 Nonattainment (2006) | −2,942.142 | −3,045.571 | −0.622 | −0.644 | 1,052.514 | 1,003.621 | −705.509 | −746.168 |
| | (1,946.370) | (2,016.899) | (0.413) | (0.422) | (868.796) | (878.862) | (578.013) | (594.524) |
| PM2.5 Nonattainment (2012) | −12,649.400*** | −11,789.730*** | −2.530** | −2.402** | −5,533.852*** | −5,209.014*** | −3,931.236*** | −3,628.948*** |
| | (1,774.609) | (1,759.863) | (1.030) | (0.974) | (944.092) | (848.677) | (528.797) | (484.270) |
| Gas/Coal Price | 2,812.477*** | 2,794.521*** | 0.752*** | 0.750*** | 983.830*** | 979.097*** | 805.311*** | 799.894*** |
| | (283.224) | (283.552) | (0.072) | (0.073) | (114.535) | (114.776) | (87.202) | (87.534) |
| Constant | 7,652.300*** | 7,675.581*** | 1.995*** | 2.011*** | 5,207.896*** | 5,235.073*** | 2,790.100*** | 2,806.324*** |
| | (884.334) | (869.851) | (0.226) | (0.223) | (361.193) | (353.245) | (273.126) | (269.503) |
| Unit FE | Yes | Yes | Yes | Yes | Yes | Yes | Yes | Yes |
| Observations | 8,664 | 8,664 | 8,664 | 8,664 | 8,664 | 8,664 | 8,664 | 8,664 |
| $R^2$ | 0.595 | 0.596 | 0.567 | 0.567 | 0.541 | 0.542 | 0.599 | 0.600 |
| Adjusted $R^2$ | 0.572 | 0.573 | 0.542 | 0.543 | 0.516 | 0.516 | 0.576 | 0.577 |

*Note:* *p<0.1; **p<0.05; ***p<0.01

Table S5: Resulting coefficient estimates after regressing each of the four unit emissions measures on new ambient $SO_2$ monitors added within 50 km of the unit since 2010. Other independent variables represent the county's $PM_{2.5}$ status with respect to the 2006 and 2012 standards and the national gas/coal unit price. For each dependent variable, the first column shows the results of equation (2) and the second column shows the results of equation (4).

| | *Dependent variable:* | | | | | | | |
|---|---|---|---|---|---|---|---|---|
| | Annual SO2 (tons) | | Annual SO2 Rate (g/kWh) | | 99%ile SO2 Hour (lbs) | | 50%ile SO2 hr (lbs) | |
| Post-2010 New Monitor Count | −1,365.774*** | | −0.362*** | | −586.616*** | | −406.045*** | |
| | (367.225) | | (0.105) | | (156.187) | | (120.192) | |
| Post-2010 New Monitor Count (R 1) | | −1,940.803*** | | −0.526*** | | −793.712** | | −591.641*** |
| | | (597.883) | | (0.196) | | (318.528) | | (187.322) |
| Post-2010 New Monitor Count (R 2) | | −1,048.052 | | −0.101 | | −331.894 | | −283.618 |
| | | (691.380) | | (0.141) | | (299.726) | | (230.930) |
| Post-2010 New Monitor Count (R 3) | | −1,545.057*** | | −0.632** | | −847.325*** | | −483.664** |
| | | (589.963) | | (0.251) | | (286.832) | | (207.446) |
| Post-2010 New Monitor # (R 4) | | 411.735 | | 0.106 | | 160.800 | | 116.576 |
| | | (710.631) | | (0.187) | | (267.784) | | (235.456) |
| PM2.5 Nonattainment (2006) | −2,901.973 | −2,882.720 | −0.578 | −0.562 | 1,071.089 | 1,088.008 | −719.841 | −712.869 |
| | (1,992.705) | (1,999.032) | (0.406) | (0.405) | (840.234) | (832.380) | (602.864) | (604.643) |
| PM2.5 Nonattainment (2012) | −13,234.520*** | −12,970.260*** | −2.724** | −2.665** | −5,786.714*** | −5,705.846*** | −4,074.466*** | −3,990.920*** |
| | (1,828.082) | (1,690.043) | (1.084) | (1.036) | (969.738) | (921.097) | (521.232) | (474.761) |
| Gas/Coal Price | 2,782.982*** | 2,773.996*** | 0.755*** | 0.746*** | 971.631*** | 962.710*** | 787.216*** | 783.583*** |
| | (292.634) | (292.259) | (0.071) | (0.071) | (113.529) | (113.504) | (88.718) | (88.610) |
| Constant | 7,512.814*** | 7,540.541*** | 1.911*** | 1.940*** | 5,146.087*** | 5,173.614*** | 2,786.334*** | 2,797.545*** |
| | (902.954) | (901.796) | (0.219) | (0.218) | (350.305) | (350.230) | (273.749) | (273.414) |
| Unit FE | Yes | Yes | Yes | Yes | Yes | Yes | Yes | Yes |
| Observations | 8,664 | 8,664 | 8,664 | 8,664 | 8,664 | 8,664 | 8,664 | 8,664 |
| $R^2$ | 0.598 | 0.599 | 0.567 | 0.571 | 0.543 | 0.544 | 0.602 | 0.604 |
| Adjusted $R^2$ | 0.576 | 0.577 | 0.543 | 0.547 | 0.518 | 0.519 | 0.580 | 0.581 |

*Note:* *p<0.1; **p<0.05; ***p<0.01

Meanwhile, tables S6 and S7 show analogous results, but when run on the unbalanced dataset. Importantly, note that this dataset does not include observations for EGUs in years that they did not produce electricity primarily using coal, rather than treat the EGUs' emissions as 0 during these years. Similarly to the results using the balanced panel, the results from the unbalanced regressions indicate that expected annual emissions decreased in association with policy implementation and post-2010 monitor addition.

One interesting difference between the results using the balanced and unbalanced panels are the emissions reductions associated with EGUs in round 2 of the 2010 $SO_2$ NAAQS implementation schedule. While the balanced panel results suggest that round 2 EGUs that remained open did not reduce emissions as much as EGUs in other counties in response to the policy, the unbalanced panel results show significant $SO_2$ reductions for round 2 EGUs in association with the policy implementation flag. This suggests that round 2 EGUs, the EGUs most likely to have been identified by the EPA as at risk of contributing to violations, may have been more likely to close down operations or switch to natural gas than to install control technologies or otherwise reduce coal operating emissions in response to the policy.

Table S6: Resulting coefficient estimates after regressing each of the four unit emissions measures on a dummy variable indicating whether the 2010 $SO_2$ NAAQS had been implemented in the unit's county. Other independent variables represent the county's $PM_{2.5}$ status with respect to the 2006 and 2012 standards and the national gas/coal unit price. For each dependent variable, the first column shows the results of equation (1) and the second column shows the results of equation (3).

| | *Dependent variable:* | | | | | | | |
|---|---|---|---|---|---|---|---|---|
| | Annual SO2 (tons) | | Annual SO2 Rate (g/kWh) | | 99%ile SO2 Hour (lbs) | | 50%ile SO2 hr (lbs) | |
| Policy Flag | −2,765.360*** | | −58.164 | | −1,176.558*** | | −738.190*** | |
| | (423.136) | | (58.673) | | (202.580) | | (127.345) | |
| Policy Flag (R 1) | | −3,290.700** | | 87.187 | | −1,113.810* | | −781.287 |
| | | (1,625.698) | | (90.824) | | (661.141) | | (482.436) |
| Policy Flag (R 2) | | −2,683.526*** | | −289.753 | | −1,012.507*** | | −653.152*** |
| | | (682.704) | | (291.196) | | (366.007) | | (219.408) |
| Policy Flag (R 3) | | −2,545.071*** | | 54.330 | | −1,337.208*** | | −780.939*** |
| | | (338.494) | | (57.363) | | (209.383) | | (99.010) |
| Policy Flag (R 4) | | | | | | | | |
| PM2.5 Nonattainment (2006) | −2,575.284* | −2,605.474* | 54.907 | 66.012 | 195.474 | 195.520 | −596.621 | −600.633 |
| | (1,404.906) | (1,438.086) | (57.954) | (69.574) | (607.275) | (612.390) | (439.586) | (446.825) |
| PM2.5 Nonattainment (2012) | −8,855.812*** | −8,717.152*** | 106.018 | 37.962 | −3,838.678*** | −3,845.636*** | −2,834.788*** | −2,819.053*** |
| | (3,430.717) | (3,287.890) | (111.713) | (58.418) | (1,368.953) | (1,391.017) | (1,046.777) | (1,035.850) |
| Gas/Coal Price | 2,024.596*** | 2,019.352*** | 40.701 | 41.056 | 623.485*** | 624.970*** | 527.952*** | 527.917*** |
| | (162.272) | (163.138) | (41.642) | (42.011) | (71.865) | (72.295) | (51.327) | (51.605) |
| Constant | 10,143.990*** | 10,136.980*** | −115.224 | −128.160 | 6,344.178*** | 6,356.504*** | 3,664.026*** | 3,668.633*** |
| | (499.200) | (489.425) | (122.370) | (135.664) | (223.905) | (218.555) | (158.598) | (156.063) |
| Unit FE | Yes | Yes | Yes | Yes | Yes | Yes | Yes | Yes |
| Observations | 17,210 | 17,210 | 15,886 | 15,886 | 17,189 | 17,189 | 17,189 | 17,189 |
| $R^2$ | 0.631 | 0.631 | 0.121 | 0.121 | 0.593 | 0.593 | 0.642 | 0.642 |
| Adjusted $R^2$ | 0.604 | 0.604 | 0.059 | 0.059 | 0.563 | 0.563 | 0.616 | 0.616 |

*Note:* *p<0.1; **p<0.05; ***p<0.01

Table S7: Resulting coefficient estimates after regressing each of the four unit emissions measures on new ambient $SO_2$ monitors added within 50 km of the unit since 2010. Other independent variables represent the county's $PM_{2.5}$ status with respect to the 2006 and 2012 standards and the national gas/coal unit price. For each dependent variable, the first column shows the results of equation (2) and the second column shows the results of equation (4).

| | *Dependent variable:* | | | | | | | |
|---|---|---|---|---|---|---|---|---|
| | Annual SO2 (tons) | | Annual SO2 Rate (g/kWh) | | 99%ile SO2 Hour (lbs) | | 50%ile SO2 hr (lbs) | |
| Post-2010 New Monitor Count | −1,287.267*** | | 22.818 | | −562.432*** | | −384.252*** | |
| | (312.101) | | (24.175) | | (146.035) | | (101.347) | |
| Post-2010 New Monitor Count (R 1) | | −1,036.645* | | 37.493 | | −444.032 | | −308.998* |
| | | (530.476) | | (39.303) | | (286.373) | | (170.380) |
| Post-2010 New Monitor Count (R 2) | | −1,446.564** | | −16.715 | | −520.458* | | −419.608** |
| | | (613.911) | | (24.717) | | (268.223) | | (204.284) |
| Post-2010 New Monitor Count (R 3) | | −1,552.611*** | | 45.808 | | −776.739*** | | −472.727*** |
| | | (459.959) | | (48.324) | | (219.142) | | (156.212) |
| Post-2010 New Monitor Count (R 4) | | −239.280 | | 42.337 | | −234.673 | | −80.077 |
| | | (682.916) | | (44.465) | | (274.584) | | (218.632) |
| PM2.5 Nonattainment (2006) | −2,392.019* | −2,377.575* | 76.270 | 73.725 | 278.782 | 290.960 | −559.943 | −555.432 |
| | (1,420.944) | (1,425.031) | (79.576) | (76.964) | (598.494) | (598.471) | (447.501) | (448.977) |
| PM2.5 Nonattainment (2012) | −9,421.963** | −9,515.792** | 82.903 | 78.417 | −4,081.359*** | −4,131.370*** | −2,985.704*** | −3,014.380*** |
| | (3,754.003) | (3,830.232) | (92.854) | (89.747) | (1,501.573) | (1,539.603) | (1,117.823) | (1,140.379) |
| Gas/Coal Price | 2,032.598*** | 2,028.220*** | 48.757 | 49.509 | 623.932*** | 619.376*** | 523.929*** | 522.391*** |
| | (170.394) | (169.442) | (49.889) | (50.659) | (73.255) | (72.915) | (53.162) | (52.872) |
| Constant | 9,828.202*** | 9,841.713*** | −146.202 | −148.522 | 6,218.949*** | 6,233.007*** | 3,598.735*** | 3,603.482*** |
| | (525.769) | (522.831) | (153.938) | (156.315) | (226.035) | (224.986) | (164.038) | (163.141) |
| Unit FE | Yes | Yes | Yes | Yes | Yes | Yes | Yes | Yes |
| Observations | 17,016 | 17,016 | 15,776 | 15,776 | 16,995 | 16,995 | 16,995 | 16,995 |
| $R^2$ | 0.630 | 0.630 | 0.121 | 0.121 | 0.595 | 0.596 | 0.642 | 0.643 |
| Adjusted $R^2$ | 0.603 | 0.604 | 0.060 | 0.060 | 0.567 | 0.567 | 0.617 | 0.617 |

*Note:* *p<0.1; **p<0.05; ***p<0.01

### 3.4 Regressions with Price Effect by EGU

The following sensitivity analysis considers the possibility that different EGUs responded differently to changes in fuel prices over time due to heterogeneity in plant characteristics, including plant location. Table S8 below shows the regression results estimating the effect of the 2010 $SO_2$ NAAQS policy flag when EGU fixed effects are interacted with the ratio of the national average natural gas price to coal price over time, while Table S8 shows the results with new monitor count as the policy measure. Both tables show that the policy measures are associated with significant $SO_2$ reductions across all measures.

The relative effects of the policy implementation flag on $SO_2$ emissions in Table S8 show that round 2 rather than round 3 EGUs had the greatest emissions reductions, but the between-round differences are only significant for rounds 2 and 3 with respect to $SO_2$ emissions rate and median $SO_2$ emissions. Meanwhile, the effect of new monitor additions in Table S9 is still most negative for round 3 EGUs.

Table S8: $SO_2$ emissions changes associated with policy implementation flag with EGU fixed effects interacted with gas/coal price.

| | *Dependent variable:* | | | | | | | |
|---|---|---|---|---|---|---|---|---|
| | log(Annual SO2 (tons)) | | log(Annual SO2 Rate (g/kWh)) | | log(99%ile SO2 Hour (lbs)) | | log(50%ile SO2 hr (lbs)) | |
| Policy Flag | −0.865*** | | −0.543*** | | −0.454*** | | −0.538*** | |
| | (0.065) | | (0.059) | | (0.049) | | (0.056) | |
| Policy Flag (Round 1) | | −0.774*** | | −0.506*** | | −0.432*** | | −0.483*** |
| | | (0.172) | | (0.127) | | (0.120) | | (0.138) |
| Policy Flag (Round 2) | | −0.960*** | | −0.690*** | | −0.502*** | | −0.684*** |
| | | (0.121) | | (0.124) | | (0.096) | | (0.113) |
| Policy Flag (Round 3) | | −0.817*** | | −0.444*** | | −0.424*** | | −0.444*** |
| | | (0.077) | | (0.060) | | (0.059) | | (0.063) |
| Policy Flag (Round 4) | | | | | | | | |
| PM2.5 Nonattainment (2006) | 0.336 | 0.359 | 0.348 | 0.368 | 0.458** | 0.466** | 0.229 | 0.251 |
| | (0.254) | (0.260) | (0.235) | (0.240) | (0.184) | (0.187) | (0.232) | (0.236) |
| PM2.5 Nonattainment (2012) | −0.596*** | −0.621*** | −0.148 | −0.158 | −0.219*** | −0.225*** | −0.058 | −0.073 |
| | (0.172) | (0.151) | (0.128) | (0.119) | (0.060) | (0.062) | (0.337) | (0.324) |
| Gas/Coal Price | 1.122*** | 1.125*** | 1.093*** | 1.101*** | 0.648*** | 0.651*** | 0.987*** | 0.995*** |
| | (0.005) | (0.006) | (0.005) | (0.005) | (0.004) | (0.005) | (0.004) | (0.005) |
| Constant | 5.625*** | 5.610*** | −2.490*** | −2.522*** | 6.857*** | 6.847*** | 5.063*** | 5.033*** |
| | (0.021) | (0.025) | (0.019) | (0.019) | (0.016) | (0.019) | (0.018) | (0.020) |
| State FE x Gas/Coal Price | No | No | No | No | No | No | No | No |
| County FE x Gas/Coal Price | No | No | No | No | No | No | No | No |
| Facility FE x Gas/Coal Price | No | No | No | No | No | No | No | No |
| Unit FE x Gas/Coal Price | Yes | Yes | Yes | Yes | Yes | Yes | Yes | Yes |
| Observations | 8,778 | 8,778 | 8,778 | 8,778 | 8,778 | 8,778 | 8,772 | 8,772 |
| $R^2$ | 0.783 | 0.783 | 0.760 | 0.760 | 0.758 | 0.758 | 0.771 | 0.771 |
| Adjusted $R^2$ | 0.758 | 0.758 | 0.732 | 0.733 | 0.730 | 0.730 | 0.744 | 0.745 |

*Note:* *p<0.1; **p<0.05; ***p<0.01

Table S9: $SO_2$ emissions changes associated with new monitor additions with EGU fixed effects interacted with gas/coal price.

| | *Dependent variable:* | | | | | | | |
|---|---|---|---|---|---|---|---|---|
| | log(Annual SO2 (tons)) | | log(Annual SO2 Rate (g/kWh)) | | log(99%ile SO2 Hour (lbs)) | | log(50%ile SO2 hr (lbs)) | |
| Post-2010 New Monitor Count | −0.292*** | | −0.205*** | | −0.164*** | | −0.206*** | |
| | (0.037) | | (0.031) | | (0.024) | | (0.031) | |
| Post-2010 New Monitor Count (Round 1) | | −0.299*** | | −0.217*** | | −0.161*** | | −0.211*** |
| | | (0.091) | | (0.066) | | (0.059) | | (0.065) |
| Post-2010 New Monitor Count (Round 2) | | −0.200*** | | −0.163*** | | −0.135*** | | −0.159*** |
| | | (0.038) | | (0.037) | | (0.029) | | (0.032) |
| Post-2010 New Monitor Count (Round 3) | | −0.400*** | | −0.263*** | | −0.219*** | | −0.258*** |
| | | (0.067) | | (0.069) | | (0.054) | | (0.068) |
| Post-2010 New Monitor Count (Round 4) | | −0.227** | | −0.092* | | −0.020 | | −0.195 |
| | | (0.101) | | (0.051) | | (0.044) | | (0.132) |
| PM2.5 Nonattainment (2006) | 0.434* | 0.436* | 0.390 | 0.389 | 0.500*** | 0.500*** | 0.268 | 0.269 |
| | (0.245) | (0.249) | (0.239) | (0.244) | (0.182) | (0.184) | (0.236) | (0.241) |
| PM2.5 Nonattainment (2012) | −0.682*** | −0.648*** | −0.192*** | −0.172** | −0.259** | −0.245* | −0.101 | −0.084 |
| | (0.075) | (0.086) | (0.069) | (0.069) | (0.118) | (0.129) | (0.238) | (0.228) |
| Gas/Coal Price | 1.190*** | 1.190*** | 1.136*** | 1.136*** | 0.684*** | 0.684*** | 1.029*** | 1.029*** |
| | (0.000) | (0.000) | (0.000) | (0.000) | (0.000) | (0.000) | (0.000) | (0.000) |
| Constant | 5.347*** | 5.347*** | −2.664*** | −2.664*** | 6.711*** | 6.711*** | 4.890*** | 4.890*** |
| | (0.000) | (0.000) | (0.000) | (0.000) | (0.000) | (0.000) | (0.000) | (0.000) |
| State FE x Gas/Coal Price | No | No | No | No | No | No | No | No |
| County FE x Gas/Coal Price | No | No | No | No | No | No | No | No |
| Facility FE x Gas/Coal Price | No | No | No | No | No | No | No | No |
| Unit FE x Gas/Coal Price | Yes | Yes | Yes | Yes | Yes | Yes | Yes | Yes |
| Observations | 8,778 | 8,778 | 8,778 | 8,778 | 8,778 | 8,778 | 8,772 | 8,772 |
| $R^2$ | 0.764 | 0.765 | 0.750 | 0.751 | 0.749 | 0.749 | 0.763 | 0.763 |
| Adjusted $R^2$ | 0.737 | 0.738 | 0.721 | 0.722 | 0.720 | 0.720 | 0.735 | 0.735 |
| *Note:* | | | | | | | | *p<0.1; **p<0.05; ***p<0.01 |

## 3.5 Regressions with Annual Fixed Effects

This section presents a sensitivity analysis that includes annual fixed effects in the estimation approach. These fixed effects were not included in the main analysis due to the nature of the implementation of the NAAQS policy. The 2010 $SO_2$ NAAQS revision affected all counties in the U.S., which limited the ability to compare EGU emissions changes against a control group of plants not affected by the policy. Furthermore, although the policy implementation was staggered, there was a correlation between plant emissions and the timing of implementation built into the design of the policy which frustrated comparison between plants that had not yet been treated by the policy and those that had. For these reasons, the main analysis is limited to a single-difference estimation comparing individual EGU behaviors before and after policy implementation and accounts for heterogeneity over time through controls on PM2.5 attainment status and gas vs. coal price. Including annual fixed effects would absorb much of the variation we are hoping to observe.

Tables S10 and S11 show the estimation results of the effects of 2010 $SO_2$ NAAQS policy implementation and new $SO_2$ monitor addition respectively on EGU $SO_2$ emissions. Table S10 shows that the effect policy implementation changes signs from negative to positive, though is only significant for $99^{th}$ percentile hourly emissions. This change in sign is consistent with the strong correlation between implementation round timing and emissions. EGUs in earlier implementation rounds would make up most of the observations post-policy implementation, and simultaneously selected into early implementation rounds due to characteristics that are plausibly related to unresponsiveness to policy. Since the addition of annual fixed effects creates comparisons between plants within years, this endogenous selection into policy round by plant characteristics results in comparisons between incomparable sets of plants.

This explanation is supported by the results in Table S11, which show that while including annual fixed effects reduces the magnitude of the effects of monitor additions on plant emissions, the estimated effect is still negative. Since the timing of additional $SO_2$ monitoring equipment was more orthogonal to plant characteristics relative to implementation round assignment, the effect of these monitor additions was less correlated with annual fixed effects. This is further helped by

the fact that each implementation round had EGUs that were and were not treated with nearby monitor additions, meaning that the effects of monitor additions within these groups of EGUs could be more plausibly compared within the same year. We also do see that the relative effect of monitor additions on emissions between the implementation rounds was most negative for round 3 EGUs, the same finding as with the main regression.

Table S10: Effects of 2010 $SO_2$ NAAQS implementation by policy round on $SO_2$ emissions with annual fixed effects.

| | *Dependent variable:* | | | | | | | |
|---|---|---|---|---|---|---|---|---|
| | log(Annual SO2 (tons)) | | log(Annual SO2 Rate (g/kWh)) | | log(99%ile SO2 Hour (lbs)) | | log(50%ile SO2 hr (lbs)) | |
| Policy Flag | 0.265* | | 0.196 | | 0.195** | | 0.200 | |
| | (0.141) | | (0.127) | | (0.094) | | (0.125) | |
| Policy Flag (Round 1) | | 0.263 | | 0.240 | | 0.139 | | 0.245 |
| | | (0.271) | | (0.201) | | (0.139) | | (0.202) |
| Policy Flag (Round 2) | | 0.392** | | 0.253 | | 0.309** | | 0.245 |
| | | (0.185) | | (0.178) | | (0.134) | | (0.175) |
| Policy Flag (Round 3) | | −0.106 | | −0.073 | | −0.009 | | −0.037 |
| | | (0.140) | | (0.093) | | (0.089) | | (0.113) |
| Policy Flag (Round 4) | | | | | | | | |
| PM2.5 Nonattainment (2006) | −0.442** | −0.450** | −0.278* | −0.280* | −0.065 | −0.074 | −0.393** | −0.393** |
| | (0.211) | (0.218) | (0.160) | (0.165) | (0.112) | (0.115) | (0.175) | (0.179) |
| PM2.5 Nonattainment (2012) | −0.035 | −0.037 | 0.204 | 0.178 | 0.052 | 0.081 | 0.316*** | 0.289* |
| | (0.201) | (0.256) | (0.300) | (0.344) | (0.156) | (0.162) | (0.112) | (0.152) |
| Gas/Coal Price | 0.421*** | 0.426*** | 0.328*** | 0.333*** | 0.167*** | 0.167*** | 0.334*** | 0.339*** |
| | (0.087) | (0.087) | (0.075) | (0.075) | (0.054) | (0.053) | (0.074) | (0.074) |
| Constant | 7.648*** | 7.651*** | −0.252 | −0.258 | 8.383*** | 8.395*** | 7.006*** | 6.999*** |
| | (0.385) | (0.382) | (0.333) | (0.333) | (0.251) | (0.245) | (0.327) | (0.327) |
| Unit FE | Yes | Yes | Yes | Yes | Yes | Yes | Yes | Yes |
| Year FE | Yes | Yes | Yes | Yes | Yes | Yes | Yes | Yes |
| Observations | 8,778 | 8,778 | 8,778 | 8,778 | 8,778 | 8,778 | 8,772 | 8,772 |
| $R^2$ | 0.692 | 0.694 | 0.631 | 0.632 | 0.684 | 0.685 | 0.657 | 0.658 |
| Adjusted $R^2$ | 0.675 | 0.677 | 0.609 | 0.611 | 0.665 | 0.667 | 0.637 | 0.638 |

*Note:* *p<0.1; **p<0.05; ***p<0.01

Table S11: Effects of new $SO_2$ monitor additions on $SO_2$ emissions with annual fixed effects.

| | *Dependent variable:* | | | | | | | |
|---|---|---|---|---|---|---|---|---|
| | log(Annual SO2 (tons)) | | log(Annual SO2 Rate (g/kWh)) | | log(99%ile SO2 Hour (lbs)) | | log(50%ile SO2 hr (lbs)) | |
| New 50 km Monitors | −0.042 | | −0.030 | | −0.025 | | −0.033 | |
| | (0.068) | | (0.066) | | (0.052) | | (0.064) | |
| New 50 km Monitors (Round 1) | | −0.085 | | −0.067 | | −0.057 | | −0.060 |
| | | (0.132) | | (0.091) | | (0.077) | | (0.093) |
| New 50 km Monitors (Round 2) | | 0.067 | | 0.021 | | 0.002 | | 0.014 |
| | | (0.136) | | (0.133) | | (0.103) | | (0.124) |
| New 50 km Monitors (Round 3) | | $-0.193^{*}$ | | −0.103 | | −0.067 | | −0.097 |
| | | (0.107) | | (0.102) | | (0.066) | | (0.100) |
| New 50 km Monitors (Round 4) | | $0.305^{**}$ | | $0.281^{**}$ | | $0.254^{***}$ | | 0.159 |
| | | (0.137) | | (0.114) | | (0.080) | | (0.166) |
| PM2.5 Nonattainment (2006) | $-0.448^{**}$ | $-0.437^{**}$ | $-0.282^{*}$ | $-0.278^{*}$ | −0.068 | −0.066 | $-0.397^{**}$ | $-0.393^{**}$ |
| | (0.216) | (0.215) | (0.166) | (0.166) | (0.117) | (0.117) | (0.181) | (0.181) |
| PM2.5 Nonattainment (2012) | −0.025 | −0.022 | 0.212 | 0.223 | 0.061 | 0.073 | $0.324^{***}$ | $0.329^{***}$ |
| | (0.153) | (0.154) | (0.257) | (0.248) | (0.118) | (0.115) | (0.095) | (0.100) |
| Gas/Coal Price | $0.408^{***}$ | $0.384^{***}$ | $0.318^{***}$ | $0.302^{***}$ | $0.158^{***}$ | $0.146^{***}$ | $0.324^{***}$ | $0.312^{***}$ |
| | (0.086) | (0.085) | (0.074) | (0.074) | (0.054) | (0.052) | (0.073) | (0.073) |
| Constant | $7.680^{***}$ | $7.786^{***}$ | −0.228 | −0.155 | $8.404^{***}$ | $8.460^{***}$ | $7.031^{***}$ | $7.085^{***}$ |
| | (0.379) | (0.376) | (0.331) | (0.330) | (0.248) | (0.241) | (0.324) | (0.324) |
| Unit FE | Yes | Yes | Yes | Yes | Yes | Yes | Yes | Yes |
| Year FE | Yes | Yes | Yes | Yes | Yes | Yes | Yes | Yes |
| Observations | 8,778 | 8,778 | 8,778 | 8,778 | 8,778 | 8,778 | 8,772 | 8,772 |
| $R^2$ | 0.692 | 0.695 | 0.630 | 0.632 | 0.683 | 0.684 | 0.656 | 0.657 |
| Adjusted $R^2$ | 0.674 | 0.678 | 0.609 | 0.611 | 0.664 | 0.666 | 0.637 | 0.638 |

*Note:* $^{*}p<0.1$; $^{**}p<0.05$; $^{***}p<0.01$

# 4 Predicting Round Inclusion

Table S12 shows the results of regressing the policy round of EGUs' counties on EGU operating characteristics and state gas over national coal price. While round 4 had no significant coefficients, several EGU characteristics were correlated with an EGU's county being included in any of the first three rounds. In particular, the odds of an EGU's county being included in rounds 1 and 2 increased as tons of $SO_2$ emitted increased while the odds of inclusion in round 3 decreased. This aligns with the EPA's criteria requiring EGUs with greater emissions to be designated in round 2, and also suggests that EGUs in round 1 counties—where there was already high-quality monitoring that allowed for classification under the hourly standard—may have already been targeted for additional policy scrutiny based on their emissions quantities. It is also worth noting that round 1 EGUs tended to have less annual production while EGU size did not significantly predict round 2 inclusion.

Inclusion in round 1 and 3 was more likely for EGUs with $SO_2$ control technologies installed, whereas control installation was associated with lower odds of round 2 inclusion. One explanation could be that EGUs without control technologies may not have faced prior policy pressure to install controls and therefore had greater emissions, qualifying their counties for inclusion in round 2.

Table S12: Within-rounds logit model on units predicting round inclusion of containing counties.

| | *Dependent variable:* | | | |
|---|---|---|---|---|
| | Round 1 | Round 2 | Round 3 | Round 4 |
| log(Annual SO2 (tons)) | 0.247*** | 0.375*** | −0.395*** | 0.048 |
| | (0.041) | (0.033) | (0.028) | (0.063) |
| log(Annual Electricity Production (kWh)) | −0.121** | −0.016 | 0.070** | −0.070 |
| | (0.047) | (0.038) | (0.033) | (0.073) |
| Gas over Coal Price | 0.069** | −0.380*** | 0.291*** | −0.061 |
| | (0.029) | (0.022) | (0.019) | (0.046) |
| Control Present Flag | 0.537*** | −1.066*** | 0.720*** | −0.204 |
| | (0.093) | (0.070) | (0.062) | (0.148) |
| Constant | −3.010*** | −2.040*** | 1.191*** | −2.265*** |
| | (0.549) | (0.417) | (0.361) | (0.797) |
| Observations | 8,714 | 8,714 | 8,714 | 8,714 |
| Log Likelihood | −2,762.283 | −4,692.875 | −5,483.900 | −1,439.179 |
| Akaike Inf. Crit. | 5,534.565 | 9,395.750 | 10,977.800 | 2,888.358 |

*Note:* *p<0.1; **p<0.05; ***p<0.01

Regression is run using the balanced dataset, i.e. all units operating continuously on coal from 2001-2019.

# 5 Violations and Enforcement

One explanation for variation in EGU emissions reductions in response to policy implementation may be noncompliance. This section presents evidence of air quality violations related to $SO_2$ for coal EGUs in the balanced dataset, and shows how these violations were distributed over time. High Priority Violations are documented in the Integrated Compliance Information System (ICIS) dataset obtainable from the ECHO program.[2] This data was filtered to only include violations involving sulfur dioxide. Facility identifiers were then matched between the ICIS data and the CAMP IDs using program identification information available from the Facility Registry Service (FRS), also made available by the EPA.[3]

In total, coal facilities in the balanced dataset were associated with 231 High Priority Violations or Federally-Reportable Violations in the ICIS dataset, with 56 occurring before 2001. Of the 174 remaining violations, 98 did not have a recorded start date, leaving 76 violations by coal plants that operated continuously from 2001-2019 that can be confirmed as beginning after 2001. Table S13 shows the number of violations incurred by coal EGUs in each implementation round of the NAAQS both out of the 174 possible post-2000 violations and the violations with listed start dates of 2001 or later. Including these undated violations changes how the relative rates of violation compare between the groups. If the analysis is limited to violations in which start dates were reported, round 2 EGUs averaged 0.16 violations per EGU compared with 0.20 violations per EGU in round 3. If all possible post-2000 violations are considered, round 2 EGUs averaged 0.47 violations per EGU compared with 0.39 violations per EGU for round 3 EGUs. It is therefore not possible to confidently compare the violation rates amongst EGUs in different 2010 $SO_2$ NAAQS implementation rounds without knowing the dates of all reported violations.

Figure S8 shows the total number of new High Priority Violations were documented each year, and whether or not those violations were explicitly related to violating state SIP requirements for $SO_2$. The number of reported violations goes down considerably after 2010, which could indicate compliance with the 2010 $SO_2$ NAAQS. However, it is not possible to draw firm conclusions without knowing the temporal distribution of violations reported in ICIS without start and end dates.

Table S13: Number of reported CAA violations pursued by national and state EPAs, both without reported dates and with reported dates after 2000.

| Round | Units | Possible post-2000 violations | Definite post-2000 violations |
|---|---|---|---|
| Round 1 | 46 | 28 | 8 |
| Round 2 | 116 | 55 | 19 |
| Round 3 | 212 | 83 | 42 |
| Round 4 | 16 | 8 | 7 |

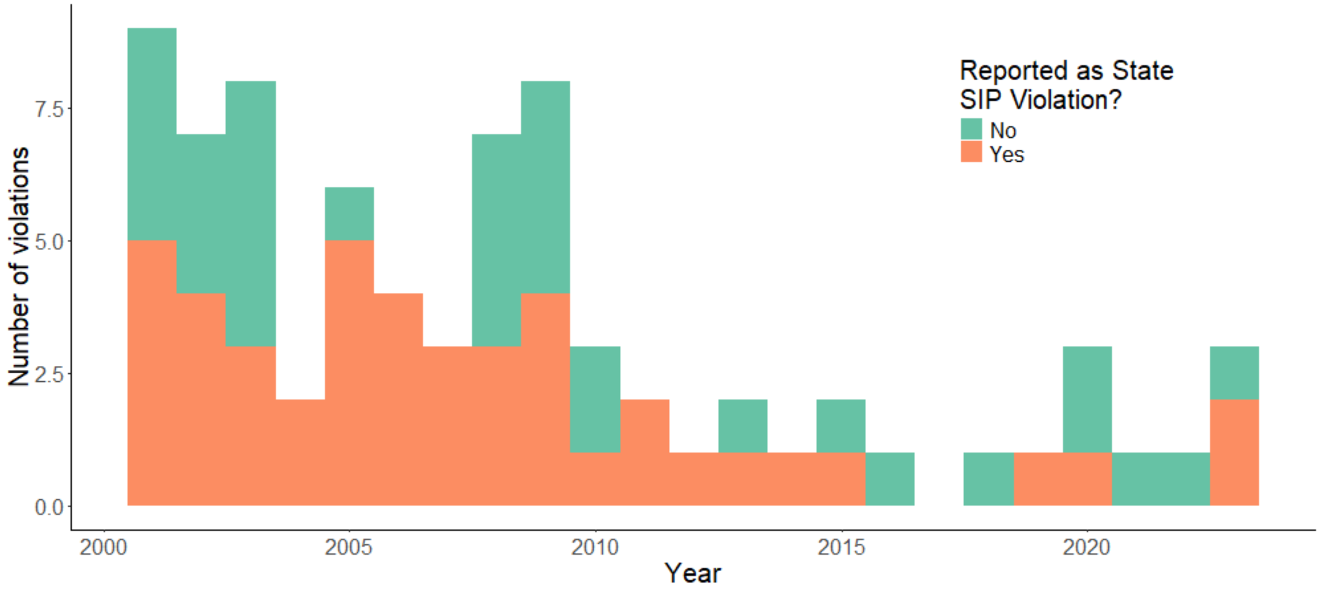


Figure S8: Annual High Priority Violations by coal plants in the balanced dataset.

This analysis shows some evidence that $SO_2$ emissions violations may have contributed to higher $SO_2$ emissions than would be expected under full compliance with the 2010 $SO_2$ NAAQS. However, the incompleteness of the ICIS data makes it infeasible to determine the true frequency of violations or the contribution of noncompliance to differences between EGU emissions reductions between rounds using this dataset alone.

## References


[1] Code of Federal Regulations. *Part 58: Ambient Air Quality Surveillance*. 1979. URL: https://www.ecfr.gov/current/title-40/chapter-I/subchapter-C/part-58 (visited on 07/28/2022).

[2] EPA. *Integrated Compliance Information System*. Jan. 2025. URL: https://echo.epa.gov/tools/data-downloads#downloads.

[3] U.S. EPA. *Facility Registry Service*. Jan. 2025. URL: https://echo.epa.gov/tools/data-downloads#downloads.
